# Spectroscopic characterization and DFT-based structural analysis of a novel sulfonyl-acetamide imidazole derivative with multi-target protein docking studies

**DOI:** 10.1039/d6ra05618k

**Published:** 2026-09-10

**Authors:** R. Geetha, V. Balachandran, R. Arulraj, N. Thirughanasambantham, B. Narayana, D. Mohan Radheep, A. Jayashree, V. S. K. Venkatachalapathy, Ponnadurai Ramasami

**Affiliations:** a Department of Physics, Arignar Anna Government Arts College (Affiliated to Bharathidasan University) Musiri Tiruchirappalli 621211 India brsbala@rediffmail.com; b Department of Physics, Sri Manakula Vinayagar Engineering College Madagadipet Puducherry 605107 India; c Department of Physics, Subasakthi College of Arts and Science for Women Sathiyamangalam, Kulithalai Karur Tamil Nadu 639 120 India; d Department of Chemistry, Sri Manakula Vinayagar Engineering College Madagadipet Puducherry 605107 India rarulraj108@gmail.com; e Department of Studies in Chemistry, Mangalore University Mangalagangotri - 574 199 India; f K.K. University Bihar Sherif Berauti Bihar 803115 India; g Department of Physics, Tamil Nadu Open University Saidapet Chennai 600015 India; h Centre for Research and Development, Sri Manakula Vinayagar Engineering College Madagadipet Puducherry 605107 India; i Computational Chemistry Group, Department of Chemistry, Faculty of Science, University of Mauritius Réduit 80837 Mauritius p.ramasami@uom.ac.mu; j Centre for Natural Product Research, Department of Chemical Sciences, University of Johannesburg Doornfontein Campus Johannesburg 2028 South Africa

## Abstract

This study describes the synthesis and thorough identification of *N*-[4-[4-[2-(2-hydroxyphenyl)-4,5-diphenyl-1*H*-imidazole-1-yl]phenyl]sulfonylphenyl]acetamide (NHDIPSA) using DFT-based computerized methods and experimental spectroscopy (liquid chromatography mass spectrometry, FT-Raman/IR, ^13^C NMR, and ^1^H NMR analysis). To evaluate its chemical reactivity, FMO and MEP surface analyses were performed, while topological studies (ELF, LOL, and NCI) elucidated the compound's intermolecular interactions. Computational PES analysis is carried out to understand the relationship between molecule energies and their geometrical structures. Vibrational and NMR assignments obtained at the B3LYP/6-311G(d,p) level showed close agreement with experimental FT-IR, FT-Raman, and NMR data, with RMSD values of 0.45–3.16 ppm for NMR chemical shifts. Docking simulations returned binding energies between −5.49 and −10.45 kcal mol^−1^ across the five target proteins, indicating favourable structural complementarity. Together, these results establish a validated structural and electronic profile for NHDIPSA and identify candidate protein targets for future experimental follow-up. No enzyme inhibition, antimicrobial, or cell-based assays were performed in this study; the biological terms used here refer to computationally predicted target compatibility, and experimental validation is left for future work.

## Abstract 2

This study describes the synthesis and identification of *N*-[4-[4-[2-(2-hydroxyphenyl)-4,5-diphenyl-1*H*-imidazole-1-yl]phenyl]sulfonylphenyl]acetamide (NHDIPSA) using DFT and spectroscopy methods (LCMS, FT-Raman/IR, ^13^C NMR, and ^1^H NMR).

## Graphical abstract

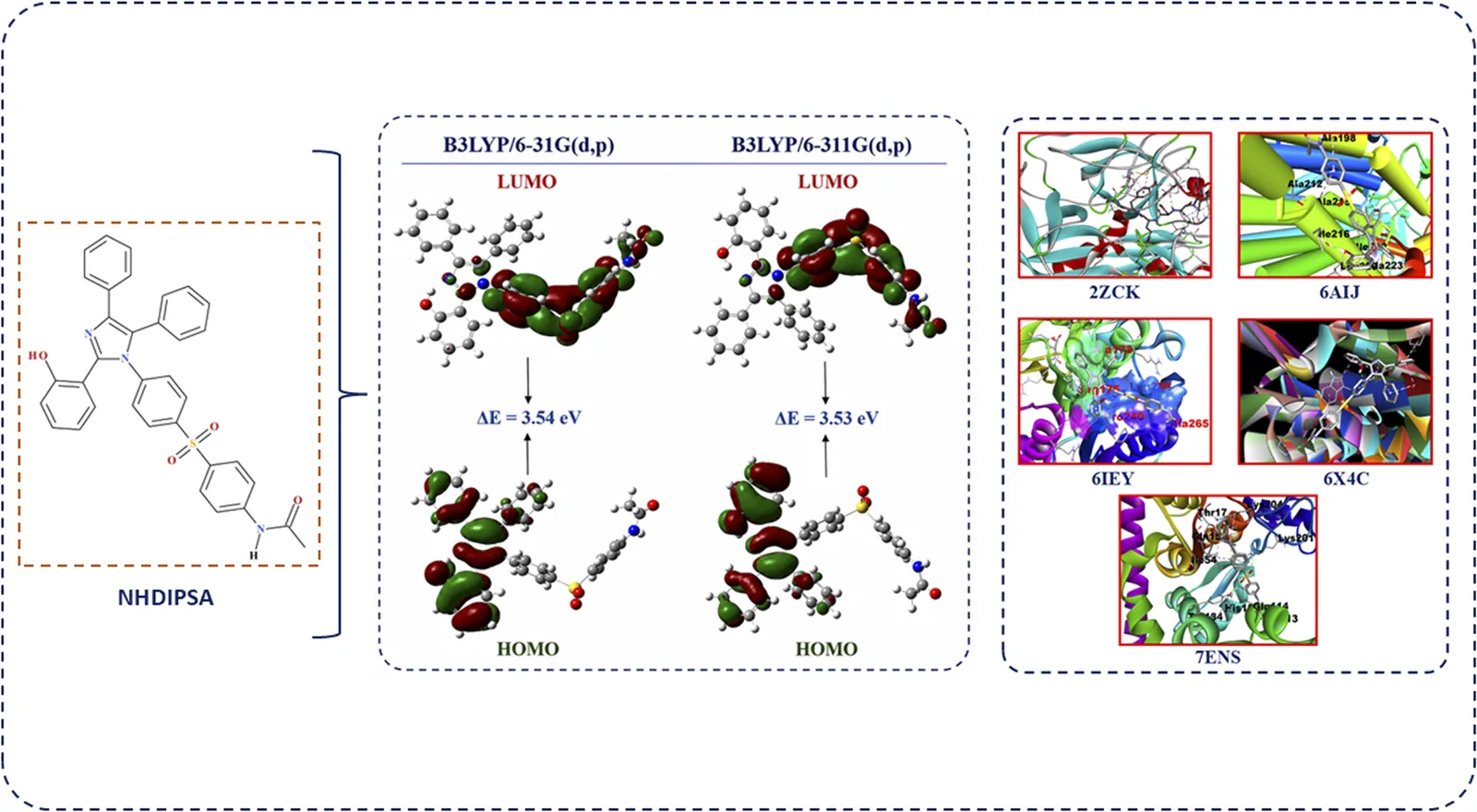

## 1 Introduction

One of the biggest risks to the successful treatment of infectious diseases globally is antimicrobial resistance (AMR), which continues to be a serious public health concern. Many current treatment medicines have become less effective due to the quick rise of resistant microbial strains, which has raised the need for novel chemical entities with better biological profiles. In medicinal chemistry, creating structurally varied heterocyclic compounds is still a key tactic for finding novel candidates that can interact with macromolecular targets that are physiologically significant.

Because of their wide range of biological activities and advantageous pharmacological characteristics, imidazole and its derivatives have a prominent position among heterocyclic systems.^1^ The imidazole ring is a five-membered aromatic heterocycle with two nitrogen atoms that give it special binding and electrical properties. The imidazole scaffold has been incorporated into several medicinal medicines and has garnered significant interest in drug development programs due to its structural versatility.^2^ Numerous imidazole compounds that are physiologically active have been documented in the literature. These substances have antihypertensive, anti-inflammatory, antifungal, antimicrobial, antiprotozoal, and anticancer properties.^3–8^ Imidazole-containing compounds have also been studied for use in the treatment of gastrointestinal problems, diabetes, malaria, and other infectious diseases. They are useful pharmacophores for the creation of bioactive chemicals because they can engage in hydrogen bonding, π–π interactions, and electrostatic contacts with biomacromolecules.

According to the current literature, no quantum computational or spectroscopic studies have included the molecule of NHDIPSA. Therefore, the purpose of this research is to address this information vacuum by combining experimental Fourier transform infrared and Fourier transform Raman spectroscopy in conjunction with Density Functional Theory (DFT) to elucidate the molecular electrical and structural properties. Current analysis used Gaussian 09W, Revision D.01 (ref. 9) and GaussView Version 6.1 (ref. 10) algorithms to forecast the most stable and optimal molecular structure. This study delves into the structure and tautomeric behaviour of the imidazole ring, a flexible scaffold that is known for its acid–base amphoteric and found in several antifungal and nitroimidazole medicines. This paper presents a thorough profile of NHDIPSA and offers new insights into its therapeutic potential by integrating theoretical modelling with biological evaluations, such as molecular docking.

As a result, this study's overarching goal is to fill this knowledge gap by investigating NHDIPSA's molecular structure, Liquid Chromatography Mass Spectrometry (LCMS), and vibrational spectrum features using Fourier transform infrared and Raman spectroscopy. We can learn more about the compound's properties and its applications from the study findings. Also investigated for this novel molecule are Potential Energy Surface (PES), Nuclear Magnetic Resonance (NMR) spectra, Molecular Electrostatic Potential (MEP), Frontier Molecular Orbitals (FMO), and topological analysis such as Electron Localization Function (ELF), Localized Orbital Locator (LOL) and Non-Covalent Interactions (NCI).

The primary objective of this study is to provide a comprehensive structural and electronic characterization of *N*-[4-[4-[2-(2-hydroxyphenyl)-4,5-diphenyl-1*H*-imidazole-1-yl]phenyl]sulfonylphenyl]acetamide (NHDIPSA) through combined experimental spectroscopy (FT-IR, FT-Raman, and NMR) and state-of-the-art Density Functional Theory (DFT) calculations. In addition, molecular docking and Prediction of Activity Spectra for Substances (PASS) analysis are employed as hypothesis-generating computational tools to explore possible ligand–target interactions with selected biological macromolecules retrieved from the RCSB Protein Data Bank,^11^ prioritized using PASS-predicted activity classes as described in Section 2.3.2. All five ligand–protein complexes examined showed favourable binding energies (−5.49 to −10.45 kcal mol^−1^) with hydrogen bonding and hydrophobic contacts at the active site, and the close match between experimental and DFT-predicted spectroscopic data confirms the identity and purity of the synthesized compound. These computational insights are intended to support structure–property correlations and to guide future experimental investigations, rather than to constitute direct evidence of pharmacological activity.

## 2 Materials and methods

### 2.1 Synthetic procedure

**Scheme 1:**
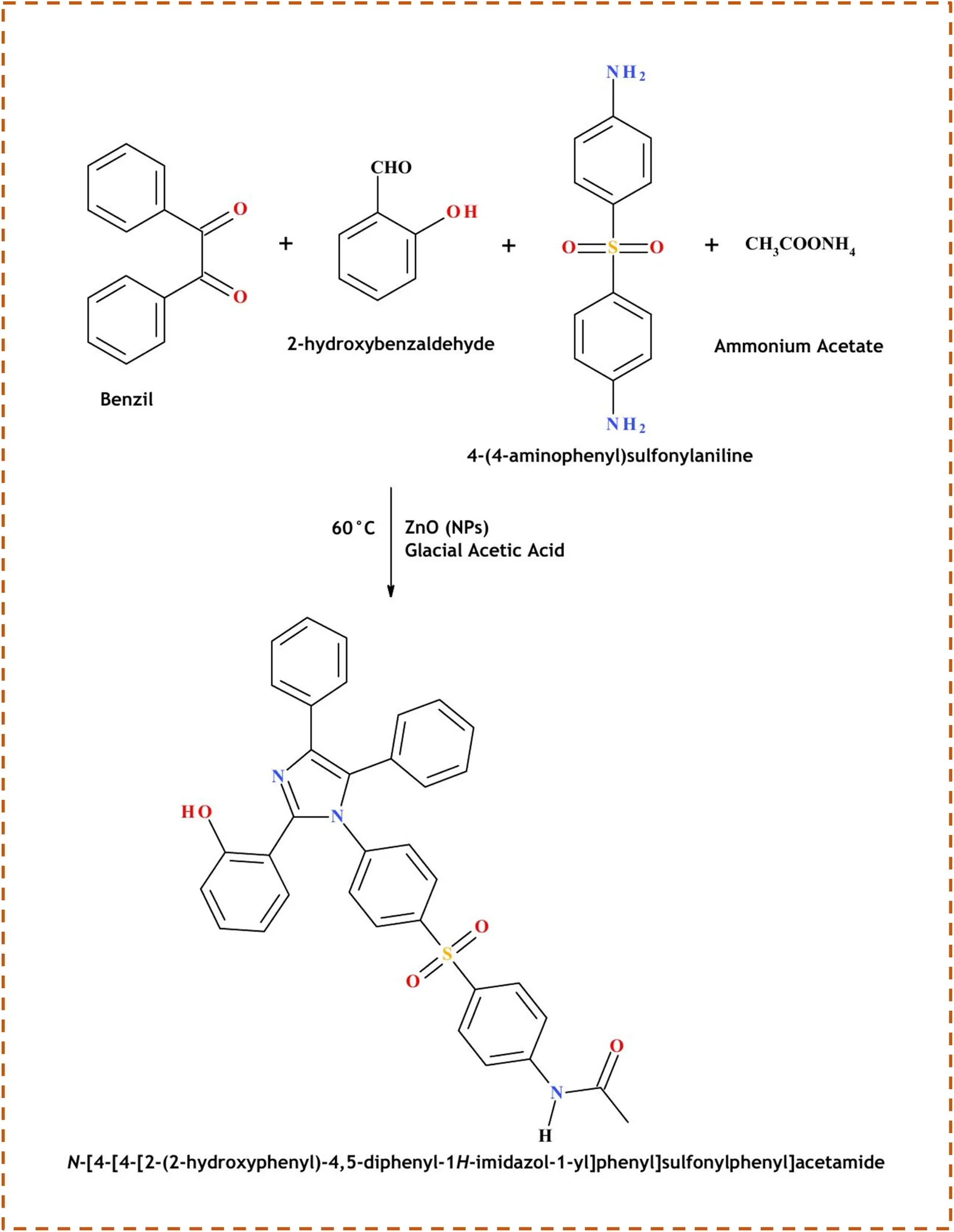
Schematic route of the synthesis of NHDIPSA.

NHDIPSA was synthesized by adding a chemical 1 : 1 mixture of benzil (1 mmol), ammonium acetate (2 mmol), 4-(4-aminophenyl)sulfonylaniline (1 mmol), and 2-hydroxybenzaldehyde (1 mmol) to glacial acetic acid (10–15 mL) using a single-pot multicomponent process. Zinc oxide (ZnO) nanoparticles (10 mol%) were added as a catalyst, and the mixture was constantly stirred at 60 °C for four to six hours. Hexane and ethyl acetate (3 : 7) as the mobile phase were used in Thin Layer Chromatography (TLC) to track the reaction's development. For the crude product to precipitate, a response liquid was brought down to room temperature, and afterwards poured over a bed of broken ice after completion. To ensure that no contaminants remained, the solid was cleansed with cold ethanol and filtered under vacuum. We used either column chromatography with the right solvent system, such as ethyl acetate/hexane, or recrystallization with ethanol to get the purified product. To get the final pure product, we used the tried-and-true Radziszewski imidazole synthesis method.^12^ The scheme shows the general flow of the synthesis of NHDIPSA (Scheme 1). SMILE structure of NHDIPSA: CC(=O)Nc1ccc(cc1)S(=O)(=O)c2ccc(cc2)n3c(nc(c4ccccc4)c3c5ccccc5)c6ccccc6O.

### 2.2 Standard spectroscopy methods

Using a KBr beam splitter, an FT-IR spectrum of the chemical was recorded using a PerkinElmer spectrum-1. The solid samples were disseminated in KBr pellets for the analysis, which covered a broad range of 4000–400 cm^−1^. A Bruker RFS 27 FT-Raman spectrometer recorded the FT-Raman spectra in the 4000–50 cm^−1^ region using an ND:YAG laser source with a wavelength of 1064 nm and 150 mw of power. The experiment was recorded in 2022 in the Sophisticated Analytical Instrumentation Facility (SAIF) at the Indian Institute of Technology (IIT) in Madras, India, which was the site of the Spectral Measurements. The ^13^C (100 MHz) and ^1^H (400 MHz) NMR (Nuclear Magnetic Resonance) spectra of NHDIPSA molecule were performed at NMR Research Centre, IISC, Bangalore, with the use of a Bruker Advance III spectrometer, with TMS serving as the internal standard and DMSO-d_6_ as the solvent. Mass spectroscopy of the compound was recorded at DST PURSE lab at Mangalore University.

### 2.3 Theoretical methodology

#### 2.3.1 DFT calculations

Based on Gaussian distribution technology (d,p) basis sets, the quantum computation was completed at DFT/B3LYP/6-31(d,p) and 6-311G(d,p). Gaussian 9.0 was the program used to observe the molecule. The same levels were used to find the improved parameters and conduct molecular surface investigations. Utilizing computational PES analysis to determine the optimal geometries of the molecule, ensuring that subsequent reactivity studies were based on its most conformationally stable state. Frequencies using the two popular techniques, Fourier transform Raman (FT-Raman) and infrared (FT-IR) and were computed along with a similar background level. The calculated wave numbers that adhere to the theoretical potential allocation of energy distribution (PED) were given individual spectral assignments using the VEDA 4 program.^13^ Applying the GIAO approach at the B3LYP/6-31G(d,p) and 6-311G(d,p) levels, the theoretical ^13^C and ^1^H NMR chemical shifts were generated. The same theoretical values were used to compute the tetramethyl silane (TMS) reference, which ensures consistency and repeatability. Incorporating solvent effects (DMSO) was done using the Integral Equation Formalism Polarizable Continuum model (IEF-PCM). To calculate chemical shifts (*δ*), the conventional relation *δ* = *σ*_TMS_ − *σ*_calc_ was used. PES scans were conducted at the B3LYP/6-31G(d,p) level, while final geometry optimizations, frequency, and property analyses were executed using the more robust B3LYP/6-311G(d,p) basis set in a PCM/DMSO solvent environment.

The previously determined basis level was used to count the HOMO–LUMO, Mullican atomic charge, and MEP. Multiwin^14^ software was employed to demonstrate the energy of the electron localization function (ELF), localized orbital locator (LOL), and reduced density gradient (RDG) in the VMD 9.1 package.^15^ A hierarchical computational strategy was adopted in this study. Potential Energy Surface (PES) scans were conducted at the B3LYP/6-31G(d,p) level to efficiently explore conformational space. Final geometry optimizations and all property-sensitive calculations, including NMR chemical shifts, Frontier Molecular Orbitals (FMO), Molecular Electrostatic Potential (MEP), and reactivity descriptors, were performed using the more robust B3LYP/6-311G(d,p) basis set to ensure enhanced polarization and electronic accuracy.

#### 2.3.2 Assessment of activity spectra (PASS) and molecular docking calculations

The online program for prediction of activity spectra for substances (PASS) was used for prediction of biochemical processes and pharmacological effects of the molecule NHDIPSA on the basis of its structural formula. The PASS online tool can predict more than four thousand types of biological function. The PASS model is able to predict adverse, toxic and pharmacological effects of the chemical NHDIPSA too. The results of PASS analysis were combined with AutoDock Tools (ADT) software version 4.2.6 to find the binding position for the functional groups of the synthesized derivative NHDIPSA. Only activities for which the predicted probability of activity (*P*_a_) exceeded the predicted probability of inactivity (*P*_i_) were retained for discussion, consistent with standard PASS interpretation practice.

The molecular docking approach for NHDIPSA was carried out using a systematic computational workflow to assess its binding affinity to specific therapeutic targets. The DFT optimised geometry of NHDIPSA was used for the ligand preparation, so as to have a structural accuracy, adding Gasteiger charges and converting to PDBQT format. Meanwhile the receptor preparation was performed by retrieving the target protein structures from RCSB Protein Data Bank, removing water molecules and co-crystallized ligands, and filling up the gaps with hydrogen atoms for stabilising protein environment using software such as PyMOL^16^ and BIOVIA Discovery Suite 4.1 (ref. 17) computer software. Once the protein's active site was centered in the grid box that defined the search area for the Lamarckian Genetic Algorithm, the docking simulations were carried out using AutoDock Vina 1.1.2.^18^ Finally, the protein–ligand complexes that were produced have a pH of 7.4 and were arranged in a ranking according to their binding affinity scores (kcal mol^−1^), and the specific interactions between molecules, including hydrogen bonding and π stacking among the imidazole scaffold and key amino acid residues, were visualized to determine the compound's inhibitory potential. The docking study was performed with five proteins obtained from RCSB Protein Data Bank^11^ and prioritized based on PASS predicted activity classes in Table 4 (PDB IDs 2ZCK, 6AIJ, 6IEY, 6X4C and 7ENS).

Molecular docking simulations were performed using the AutoDock Vina 1.1.2 software tool. The crystal structures of the target proteins 2ZCK, 6AIJ, 6IEY, 6X4C and 7ENS were obtained from the RCSB Protein Data Bank. The proteins were prepared using AutoDock Tools (ADT). ADT was used to remove co-crystallized ligands and water molecules, followed by the addition of polar hydrogens and Kollman charges. The NHDIPSA ligand was optimized at the B3LYP/6-311G(d,p) theoretical level and converted to PDBQT format with all rotatable bonds set as flexible. First, blind docking was performed to identify possible binding pockets and then a site specific docking method was used. The grid box was centered on the active site residue of each protein and was generally 126 × 126 × 126 Å^3^ with a grid spacing of 0.375 Å. The protonation state of NHDIPSA was assigned at a physiologically relevant pH (7.4). The imidazole moiety was modeled in its neutral form, and all other functional groups were treated according to their dominant protonation states under physiological conditions. The reported binding affinities are derived from AutoDock Vina scoring and do not include advanced energetic refinement such as MM/GBSA. These results therefore represent preliminary estimates intended for screening purposes.

Docking simulations were performed using AutoDock Vina with an exhaustiveness of 8 and a maximum of 10 binding poses per run. Convergence was ensured by performing three independent runs of docking with different random seeds. The best binding poses of each run showed hardly any difference in binding energy (±0.2 kcal mol^−1^), suggesting that the results are reproducible. The representative docking results from one run are summarized in Table 5. The simulation of the docking was performed using a flexible-ligand/rigid-protein model. The best-posed conformation was selected based on the lowest binding affinity (kcal mol^−1^) to study hydrogen bond and hydrophobic interactions and visualized using PyMOL and Exploration Studio Visualizer. It must be emphasized that molecular docking is an exploratory computational approach and does not take into account the protein flexibility, solvent dynamics or entropic contributions to binding. Therefore, the docking scores reported here should be interpreted qualitatively, not quantitatively. The aim of the results is to identify plausible binding modes and interaction patterns that could be the basis for further molecular dynamics simulations and experimental validation studies. The protein targets selected (PDB IDs: 2ZCK, 6AIJ, 6IEY, 6X4C and 7ENS) were based on their reported relevance in metabolic regulation, microbial pathogenicity and infectious disease mechanism in the literature. PASS predictions were used as a complementary tool for prioritization, not as the only criterion for target selection.

## 3 Results and discussion

### 3.1 Molecular structure and geometrical analysis

**Fig. 1:**
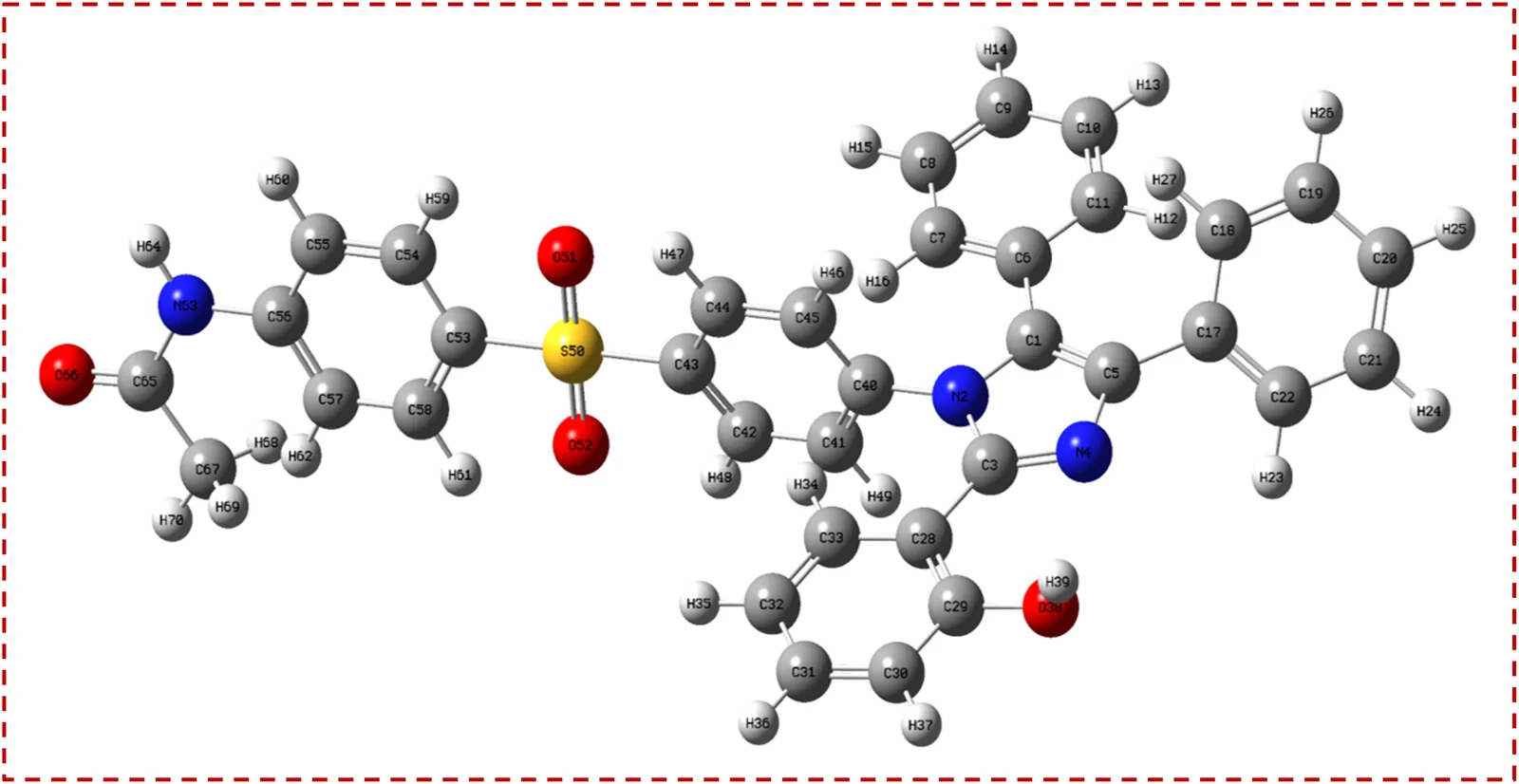
Optimized structure of NHDIPSA.

The molecular geometry of NHDIPSA optimized framework contains imidazole and aromatic benzene rings as shown in Fig. 1. An imidazole ring with phenyl groups attached is at the centre of the molecule. The five-membered imidazole ring is substituted with two nitrogen atoms, phenyl and phenylsulfonyl groups. Two phenyl rings are linked together by a sulphonyl group (–SO_2_). The theoretical and experimental geometrical parameters of NHDIPSA, including dihedral angles, bond lengths, and bond angles, are compared in Table S1. This compound contains 35 carbon–carbon bonds, 27 carbon–hydrogen bonds, 2 carbon–nitrogen bonds, 4 nitrogen–carbon bonds, 2 sulphur–oxygen bonds, 1 carbon–sulphur bond, 1 oxygen–hydrogen bond, 2 hydrogen–oxygen bonds, and 1 nitrogen–hydrogen bond, together with a series of dihedral angles that define the molecule's non-planar torsional geometry. The experimental values of carbon–carbon length of bond were found to be in the range of 1.410–1.378 Å.^19–21^

Lengths of carbon–carbon bonds of phenyl rings determined analytically, with B3LYP/6-31G(d,p) and 6-311G(d,p) methods, are in agreement with the above ranges and are C28–C33 = 1.41/1.41 Å, C5–C17 = 1.47/1.47 Å, C54–C55 = 1.39/1.39 Å. The theoretical results show that the C–H bond widths of the following compounds: C41–H49, C44–H47, C45–H46, C54–H59, C55–H60, and C57–H62, were in the range of 1.08–1.09 Å, respectively. For this situation the bond lengths are: the bond lengths C1–N2 = 1.42/1.42 Å, C56–N63 = 1.41/1.41 Å, C3– N4 = 1.34/1.34 Å and C5–N4 = 1.39/1.39 Å are computed. The bond length of the carbon–hydrogen and carbon–oxygen bonds are heteronuclear whereas the carbon–carbon bonds are homonuclear with longer bond lengths.

The difference is due to the balance of attractive and repulsive electrostatic interactions between the electron pairs of the bonds and it favors longer bond lengths for homonuclear compared to heteronuclear bonds. The calculated values of bond lengths in the present study are C43–S50 = 1.88/1.89 Å, N2–C44 = 1.44/1.44 Å, S50– O51 = 1.65/1.65 Å, S50–C53 = 1.87/1.88 Å and O38–H39 = 1.01/1.00 Å. Most of the bond angle values of this molecule which contains three phenyl rings are close to 120° which is in agreement with sp^2^ hybridization and specifically in range of 119° to 120°. The angle C9–C8–H15 is 120.09°/120.10°, the angle H68–C67–H69 is 108.33°/108.28°, the angle C43–S50–O51 is 106.16°/105.86°, and the angle N2–C3–N4 is 108.70°/108.71°. The angle created by four separate atoms in a Newman projection is known as the angle of dihedral or torsional angle. The title compound contains both positive and negative integer values for the dihedral angle. Utilizing B3LYP/6-31G(d,p) and 6-311G(d,p), the largest positive dihedral angle values are H14–C9–C10–C1 = 179.95°/179.87° and C7–C6–C11–C12 = 179.76°/179.70°. The lowest negative dihedral angle values are C6–C7–C8–H15 = −179.64°/−179.57° and C8–C9–C10–H13 = −179.92°/−179.87°.

According to the theoretical calculations at the B3LYP/6-31G(d,p) and 6-311G(d,p) levels, the sulfur–oxygen, carbon–hydrogen, carbon–nitrogen, carbon–carbon, and carbon–oxygen bond lengths of NHDIPSA fall within the expected ranges, with carbon–carbon bonds consistently longer than the heteronuclear bonds. While the torsional (dihedral) angles show both positive and negative values at ±180°, confirming planarity and stable conformations, the majority of bond angles, particularly in the phenyl rings, are close to 120°, which is consistent with sp^2^ hybridization.

### 3.2 Mass spectral analysis

Mass spectrometry reveals considerable differences in the phenyl rings. It has a molecular mass of 586.20 g mol^−1^ and chemical formula of C_35_H_27_N_3_O_4_S. The mass spectrum of NHDIPSA exhibits several peaks at *m*/*z* 586.20, 587.15 and 588.20. Molecular ion [M^+^]: the molecular ion at *m*/*z* 585.17 is the whole neutral molecule. The peak at *m*/*z* 586.20 is assigned to the protonated molecular ion [M^+^ + H], which is common in electrospray ionization (ESI) and other soft ionization techniques. The other peaks at *m*/*z* 587.15 and 588.20 are due to isotopic contributions, types of adducts or further protonation. Sodium [M + Na]^+^ and potassium [M + K]^+^ adducts were also detected at *m*/*z* values shifted by +22 and +38, respectively. Prominent fragment ions included [M + H − 59]^+^ (loss of an acetamide group) and [M + H − 64]^+^ (loss of SO_2_), confirming cleavage at the amide and sulfonyl linkages. Low-mass signals at *m*/*z* 91 and *m*/*z* 77 correspond to the tropylium and phenyl cations generated by aromatic cleavage. Sulphur (S) has a natural abundance of ∼4.2% of the isotope 34S, leading to a distinct isotopic distribution. Conversely, oxygen and nitrogen do not produce significant [M + 1] or [M + 2] isotope peaks. The [ M + 1] envelope is due to carbon-13 at about 1.1% per carbon. The molecule contains two hydrogen bond donors and four hydrogen bond acceptors which may lead to hydrogen bonding in chemical or biological systems. Its elemental composition is 71.78% carbon, 4.65% hydrogen, 7.17% nitrogen, 10.93% oxygen, and 5.47% sulphur, indicative of a carbon- and oxygen-rich framework that influences chemical reactivity, solubility, and intermolecular interactions. The evidence is thus explicitly shown in distinctive M and M + 2 peaks in the mass spectra of NHDIPSA. The fragmentation pattern well supports the proposed structure as shown in Fig. S1.

### 3.3 Potential energy surface (PES) analysis

**Fig. 2:**
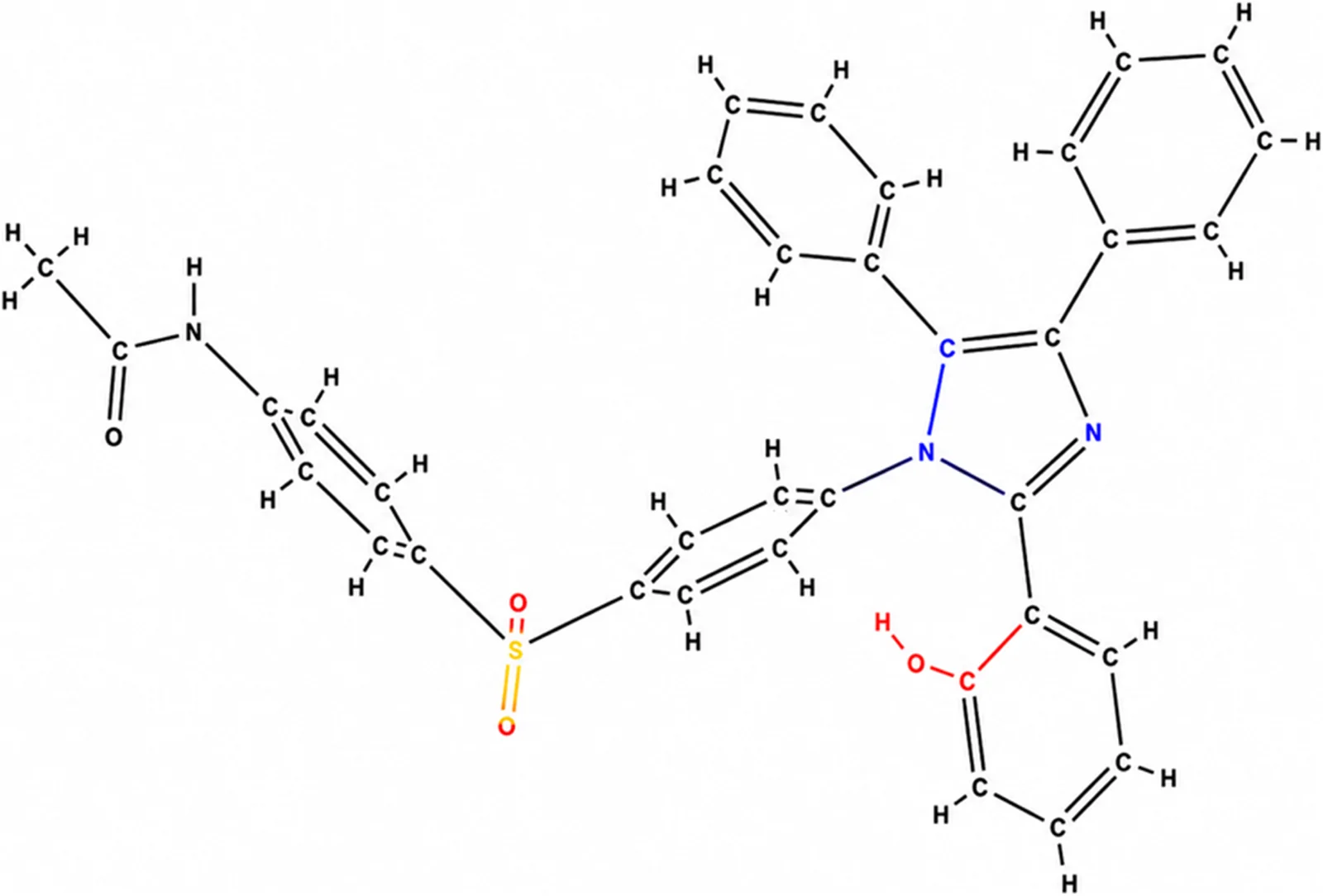
2D-configuration of NHDIPSA.

The 2D structure and the best optimized configuration of NHDIPSA are illustrated in Fig. 2. To explore the steady stable and unstable states of the molecule's, a DFT/B3LYP computational approach was employed, as it effectively characterizes molecular geometry, charge distribution, and electronic properties.^22,23^ Energy mapping was performed using the B3LYP/6-31G(d,p) and 6-311G(d,p) sets of bases, while the potential energy scan was applied to examine optimal conformations of organic compounds.^24^ The relationship between minimum energy states and molecular geometry was analyzed by systematically scanning the coordinates.^25^ NHDIPSA contains five distinct functional groups: an amide (which includes the carbonyl moiety), sulfonyl, hydroxyl, imidazole ring, and aromatic rings.

**Fig. 3:**
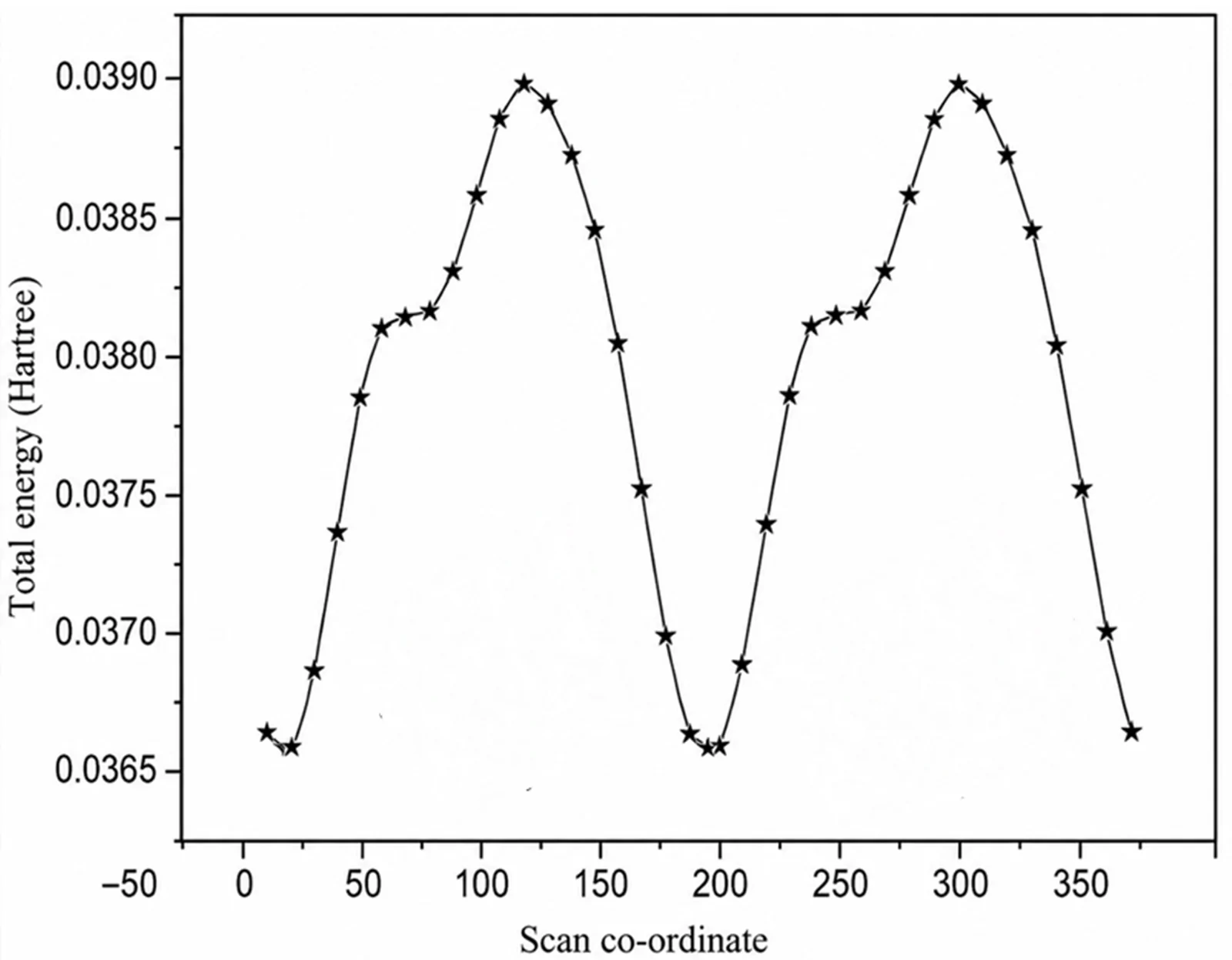
Energy function of NHDIPSA.

The conformational study was concentrated especially on the amide part, which was linked to the imidazole ring not directly, but through the intervening sulfonylphenyl linkage. During the analysis, all geometric parameters were relaxed, while specifically rotating the N4–C5–C17–C22 dihedral angle in 10° increments from 0° to 360°. The potential energy curve (Fig. 3) indicates two stable conformations at 0° and 360°. The diagram relates the dihedral rotation and the energy values (in Hartree). In the energy profile two higher energy peaks are also observed at ∼130° and ∼300° corresponding to the least stable conformers. In general, NHDIPSA has two stable rotational isomers, which are defined by *C*_2v_ symmetry. The conformation on the *Y*-axis has a potential energy of 0.0367 Hartree, whereas the conformation on the *X*-axis has a potential energy of 0.0366 Hartree and is the predominant conformer for Rot-II. The torsional flexibility at the dihedral angle between the nitrogen atom and a carbon is a key determinant of the molecule's reactivity and binding orientation, as it is particularly significant because it influences steric impacts, electronic relationships, molecular detection, and conformational stability. This, in turn, determines the molecule's structure, reactivity, and behaviour.

### 3.4 Mulliken atomic charge

The Mulliken electron charge is widely employed in quantum chemical calculations of molecular systems.^26^ This technique can be used to analyze and predict reactive activities of electrophilic and nucleophilic nature of wide variety of chemical make-ups and charge configurations in the molecule. NHDIPSA provides some interesting insights into the distribution of atomic charges. Specifically, seven atoms of carbon (C1, C3, C6, C17, C29, C56 and C65). Twenty-seven atoms of hydrogen and one atom of sulphur show a strong positive charge, three atoms of nitrogen, twenty-four atoms of carbon and four atoms of oxygen show a negative charge. These observations shed light on the delocalization direction and highlight the sensitivity of natural atomic energies to alterations in molecule structure. All hydrogen atoms are uniformly positively charged, but the carbon molecules in the ring exhibit both positive and negative energy, indicating their strong susceptibility to the action of substituents. The most negatively charged nitrogen atoms of amino groups were N2 (−0.94), N63 (−0.83) and N4 (−0.46) and the greatest positive charge was presented by the sulphur atom S50 at 1.38. Sulphur atoms were the most positively charged with the highest positive charge values, with a projected S50 = 1.38.

**Fig. 4:**
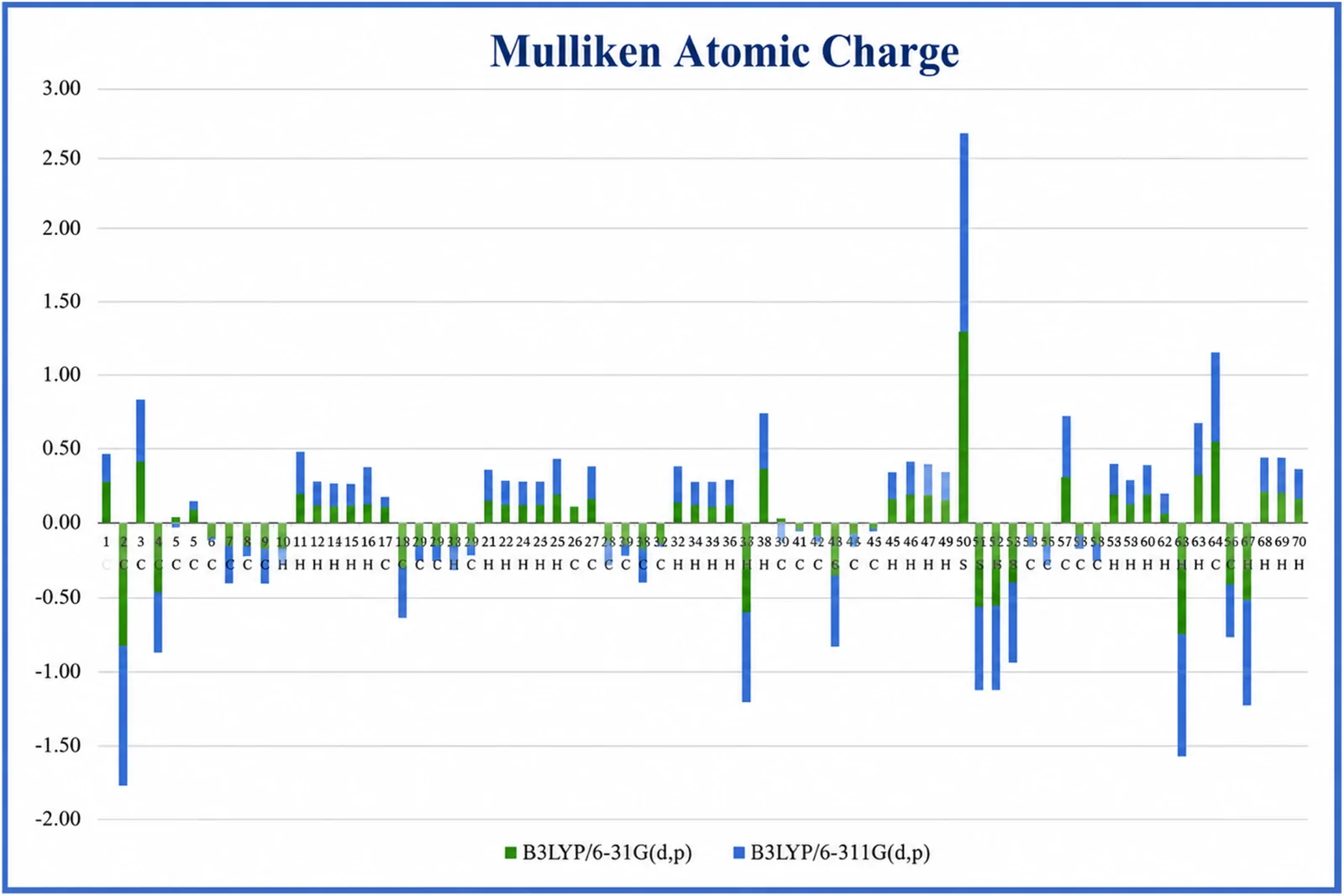
Mulliken atomic charges of the NHDIPSA.

The value of the positive charge of the hydrogen atom was H = 0.2. Seven carbon atoms are positive, C1 = 0.28, C3 = 0.42, C6 = 0.11, C17 = 0.09, C29 = 0.21, C56 = 0.39, C65 = 0.60. The carbon level in the negative regions depends on the molecules around it. These neighbour atoms have different carbon negative values. Example, C7 has a value of −0.10, C10 has a value of −0.23, C11 has a value of −0.18 *etc.* Values range from −0.69 to −0.07. The oxygen atoms in the region have different electron densities, with O38 having a value of −0.59, O51 and O52 having a value of −0.55, and O66 has a value of −0.41. Oxygen is very electronegative so it has a strong pull on the electrons and there is a strong negative charge on the oxygen atom. In contrast, electron-deficient atoms such as sulphur (S) and carbon in carbonyl groups (C=O) are often partially positive. In general, high electronegativity elements, such as oxygen (O) and nitrogen (N), bear negative charges. Fig. 4 shows the most positively and negatively charged regions of the NHDIPSA molecule and the charge distribution of the NHDIPSA molecule is presented in Table 1.

**Table 1:** Atomic charge distribution of NHDIPSA

Atoms	B3LYP/6-31G(d,p)	B3LYP/6-311G(d,p)	Atoms	B3LYP/6-31G(d,p)	B3LYP/6-311G(d,p)
1C	0.28	0.19	29C	0.17	0.21
2N	−0.82	−0.94	30C	−0.12	−0.16
3C	0.42	0.41	31C	−0.12	−0.10
4N	−0.46	−0.40	32C	−0.15	−0.23
5C	0.04	−0.03	33C	−0.13	0.01
6C	0.11	0.03	34H	0.15	0.23
7C	−0.10	0.01	35H	0.13	0.14
8C	−0.16	−0.23	36H	0.13	0.15
9C	−0.11	−0.10	37H	0.13	0.16
10C	−0.16	−0.23	38O	−0.59	−0.59
11C	−0.18	−0.12	39H	0.37	0.37
12H	0.21	0.27	40C	0.05	−0.11
13H	0.13	0.14	41C	−0.05	0.00
14H	0.12	0.14	42C	−0.07	−0.06
15H	0.12	0.14	43C	−0.36	−0.47
16H	0.14	0.23	44C	−0.07	−0.06
17C	0.09	0.07	45C	−0.05	0.00
18C	−0.29	−0.31	46H	0.16	0.17
19C	−0.12	−0.13	47H	0.20	0.21
20C	−0.12	−0.13	48H	0.20	0.21
21C	−0.14	−0.17	49H	0.17	0.18
22C	−0.12	−0.09	50S	1.30	1.38
23H	0.16	0.18	51O	−0.55	−0.54
24H	0.13	0.15	52O	−0.55	−0.54
25H	0.13	0.15	53C	−0.39	−0.54
26H	0.13	0.15	54C	−0.08	−0.04
27H	0.20	0.23	55C	−0.14	−0.14
28C	0.12	−0.03	56C	0.32	0.39
57C	−0.08	−0.09	64H	0.33	0.34
58C	−0.13	−0.12	65C	0.55	0.60
59H	0.20	0.20	66O	−0.41	−0.35
60H	0.14	0.17	67C	−0.51	−0.69
61H	0.20	0.19	68H	0.22	0.23
62H	0.08	0.12	69H	0.22	0.23
63N	−0.74	−0.83	70H	0.17	0.20

### 3.5 Nuclear magnetic resonance

Nuclear magnetic resonance (NMR) is one of the most important tools in the study of physicochemical and biological features of substances.^27^ Theoretical spectra were calculated in DMSO solvent at the DFT/B3LYP level with help of 6-31G(d,p) and 6-311G(d,p) basis sets by GIAO approach. The Tetramethylsilane (TMS) reference was computed at the same theory levels and basis sets as the NHDIPSA molecule to ensure exact consistency and repeatability. Solvent effects were explicitly included for both the TMS reference and the NHDIPSA using the IEF-PCM model and Dimethyl Sulfoxide (DMSO) as the medium. We used the conventional relation, *δ* = *σ*_TMS_ − *σ*_calc_, to obtain the theoretical chemical shifts (*δ*), where *σ* stands for the isotropic shielding constants.

The ID-theoretical as well as experimental ^1^H and ^13^C NMR spectrum of the NHDIPSA is depicted in Fig. S2 and S3. When it comes to organic compounds, the identification of NHDIPSA generally exhibits a spectrum of chemical shifts range exceeding 100 (Fig. S4). Table S2 conveys the experimentational and computational chemical variations for ^13^C and ^1^H NMR, expressed as parts per million (ppm). The experimental ^1^H and ^13^C NMR chemical shifts were observed in the ranges of 2.07–11.72 ppm and 24.15–169.23 ppm, respectively. The chemical shifts of ^1^H that were computed in the theoretical analysis using the B3LYP/6-31G(d,p) basis set (1.94–12.78 ppm) showed a better agreement with the experimental results than those that were obtained using the B3LYP/6-311G(d,p) basis set (1.00–12.00 ppm). The theoretical ^13^C NMR chemical shifts in DMSO solvent phase were in the range of 28.37–176.55 ppm (6-31G(d,p)) and 17.78–175.67 ppm (6-311G(d,p)) in comparison to the isotropic shielding of TMS calculated at the same theoretical level. The results are in good agreement with the experimental ^13^C range of 24.15–169.23 ppm. The minor discrepancies in the downfield region are justified by the solvent induced deshielding effects of the aromatic and carbonyl carbons in the IEF-PCM model.

Experimentally, in DMSO-d_6_, the NHDIPSA methyl (CH_3_) protons were detected as a singlet at 2.07 and 2.49 ppm. The chemical shifts calculated by the GIAO/B3LYP method in the IEF-PCM/DMSO solvent phase, using the 6-31G(d,p) basis set, were very accurate and ranged from 1.94 to 2.59 ppm. However, the 6-311G(d,p) basis set predicted a range of 1.00–2.00 ppm. The performance of these aliphatic protons with the basis set 6-31G(d,p) is better than the standard TMS routinely calculated indicating it accounts for the local electronic shielding in the DMSO medium.

The aromatic proton chemical changes that were found in the experiment ranged from 6.59 to 7.82 ppm. The ranges of 6.16–7.99 ppm (6-31G(d,p)) and 6.00–7.00 ppm (6-311G(d,p)) were obtained by computational analysis utilizing the GIAO/B3LYP technique in the IEF-PCM/DMSO solvent phase, respectively. The O–H group showed a theoretical prediction of 12.78 ppm and 12.00 ppm for the basis set levels, while the experimental shift for this group was 11.26 ppm which is in good agreement with these expectations. The carbonyl carbon (C65) appeared at 169.23 ppm in the ^13^C NMR spectrum and was experimentally identified. These calculations were done in the DMSO solvent phase and predicted this signal to be at 176.66 ppm (6-31G(d,p)) and 175.67 ppm (6-311G(d,p)) respectively. The theoretical values show a slight downfield shift due to the failure of the continuum solvent model to account for specific intermolecular hydrogen bonding between carbonyl oxygen and the DMSO medium. In addition, the experimental shift of 24.15 ppm for the methoxy ring carbon (C67) was in agreement with the computed values of 28.37 ppm and 17.78 ppm. A concentration of 144.69 ppm was used to identify methylene carbon (C3). All theoretical shifts were calculated relative to a TMS reference calculated at the same levels of theory and in the same solvent conditions.

The structure of the newly synthesized compound (NHDIPSA) was confirmed by detailed analysis of experimental and theoretical NMR spectra, in particular ^1^H and ^13^C-NMR. Importantly, the results show that the predicted shifts for major functional groups, including the CH_3_ group, aromatic protons, O–H group and carbonyl moiety are very close to the actual values, which is consistent with the expected molecular geometry. The changes of aromatic protons (6.59–7.82 ppm) and methyl protons (2.07–2.49 ppm) observed were perfectly reproduced by the theoretical model. The carbonyl and methoxy ring carbons exhibit good correlation between theoretical and actual signals with the former being 169.23 ppm and the latter 176.66 ppm. The good agreement between the experimental and theoretical resonance spectra is a confirmation of the structural identity of the synthesized molecule, which is also supported by the uniform application of the solvent model to both the ligand and the reference. This alignment in support of the validity of the proposed molecular architecture can be further confirmed by X-ray crystallization or mass spectrometry in future studies.

The Root Mean Square Deviation (RMSD) is a standard statistical measure used in chemistry and bioinformatics to quantify the average distance between atoms (usually between a predicted “theoretical” structure and an “experimental” structure).

The equation for calculating the RMSD is as follows (1):1
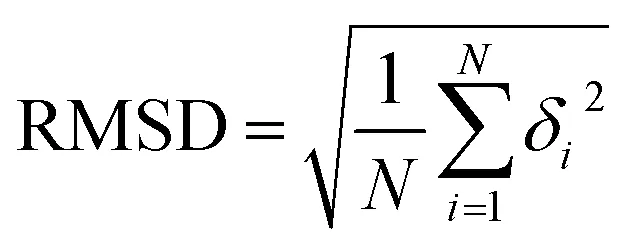
where: *N*: total number of atoms or data points being compared. Σ: the sum of the values. *δ*_*i*_: the distance (deviation) between the *i*th experimental point and the *i*th theoretical point (calculated as *δ*_exp_ − *δ*_theo_).

The statistical analysis by Root Mean Square Deviation (RMSD) has confirmed the structural integrity of NHDIPSA. The statistical results show that the B3LYP/6-31G(d,p) basis set in the PCM/DMSO phase has better accuracy for proton chemical shifts with the RMSD of 0.45 ppm instead of 1.03 ppm for the 6-311G(d,p) set. The ^13^C NMR spectra were well reproduced by both methods. The 6-311G(d,p) basis set gave a RMSD of 3.10 ppm and the 6-31G(d,p) basis set a RMSD of 3.16 ppm. Even if the carbonyl region (C65) is affected by the solvent-induced deshielding effects in the DMSO media, the overall RMSD values are still far below the threshold recognized by the science for complex heterocyclic scaffolds. Strong evidence for the effectively synthesized molecular architecture is provided by the excellent linear correlation between the experimental results and theoretical expectations. The consistency indicates that the GIAO/DFT method is a useful tool for structural characterization of imidazole derivatives when a consistent solvent model and internal reference are used.

**Fig. 5:**
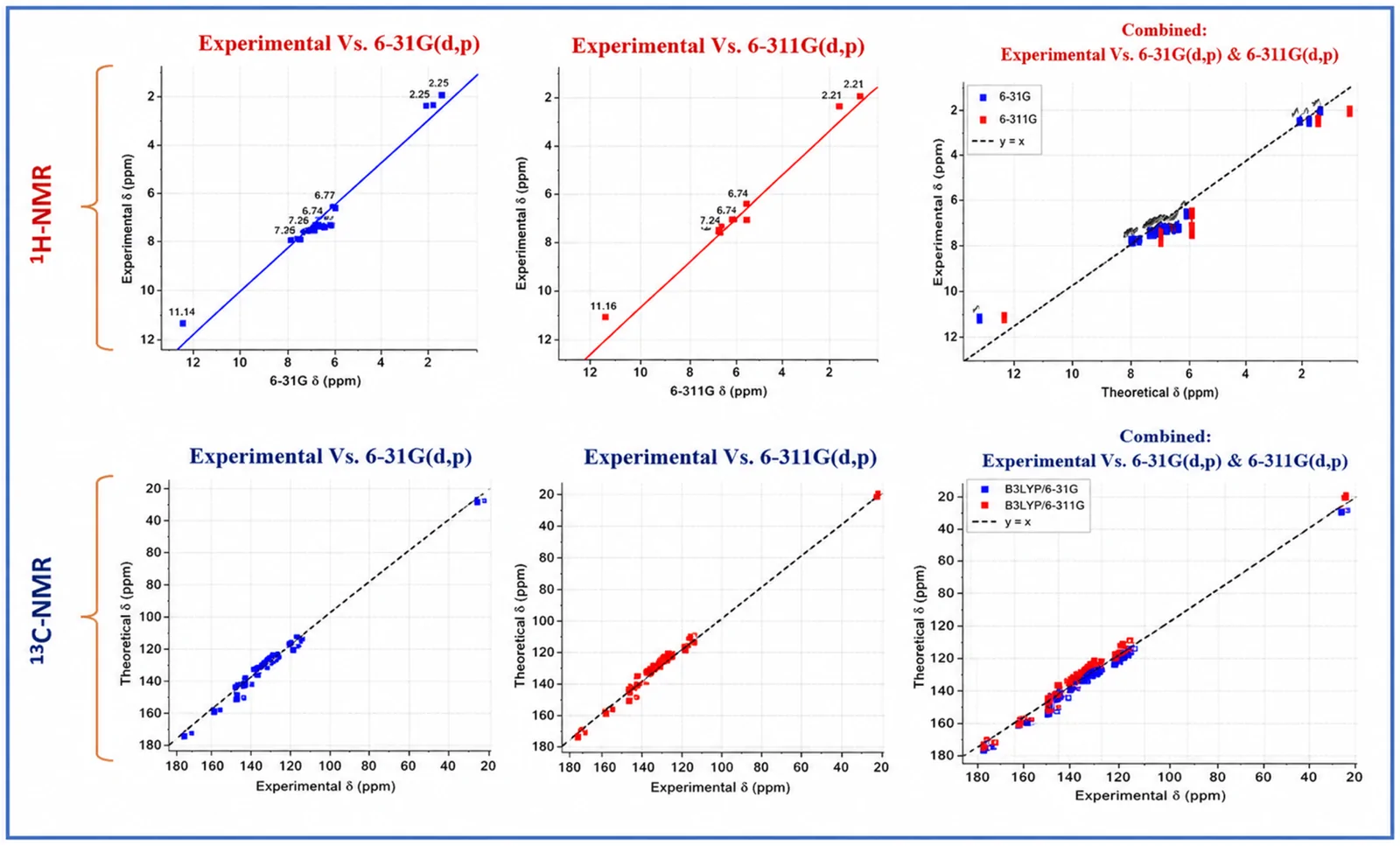
2D-experimental and theoretical ^1^H-NMR and ^13^C-NMR correlation for (B3LYP/6-31G(d,p)) and (B3LYP/6-311G(d,p)) of NHDIPSA.

Experimental ^1^H and ^13^C NMR chemical shifts are quite well reproduced by both basis sets (6-31G(d,p) and 6-311G(d,p)). Excellent linear correlation between both basis sets and experimental data is shown (*R*^2^ = 0.998). For the ^13^C, they do the same. There are variances for some carbons (67C, 57C, 35C). Both sets give good correlations for ^1^H, but in this data set 6-31G (*R*^2^ = 0.961) is better than 6-311G(d,p) (*R*^2^ = 0.943). 2D-experimental and conceptual ^1^H NMR and ^13^C NMR chemical modifications, DFT (B3LYP) computations are reliable methods for predicting NMR shifts, and 6-31G(d,p) is more economical and sometimes even more precise (Fig. 5).

Minor deviations between experimental and theoretical chemical shifts may arise from limitations of the PCM solvent model, which accounts for bulk dielectric effects but neglects explicit solute–solvent hydrogen bonding. Moreover, the NHDIPSA backbone is rigid, which should lead to small conformational averaging effects. The GIAO approach describes anisotropic shielding contributions from aromatic rings well, which is supportive of the reliability of the theoretical–experimental correlation.

### 3.6 Functional group and vibrational band assessment

**Fig. 6:**
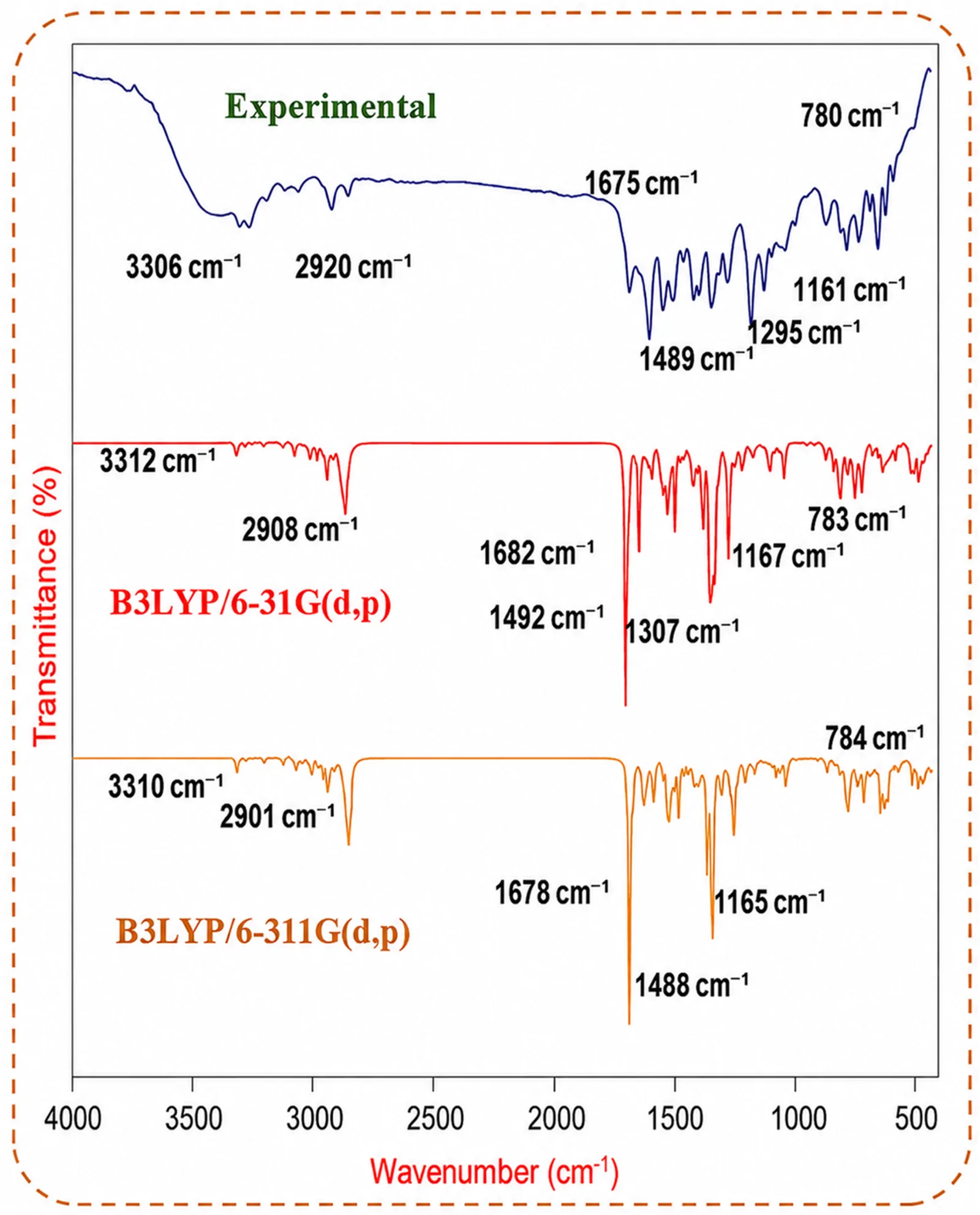
Experimental and calculated FT-IR spectra of NHDIPSA.

**Fig. 7:**
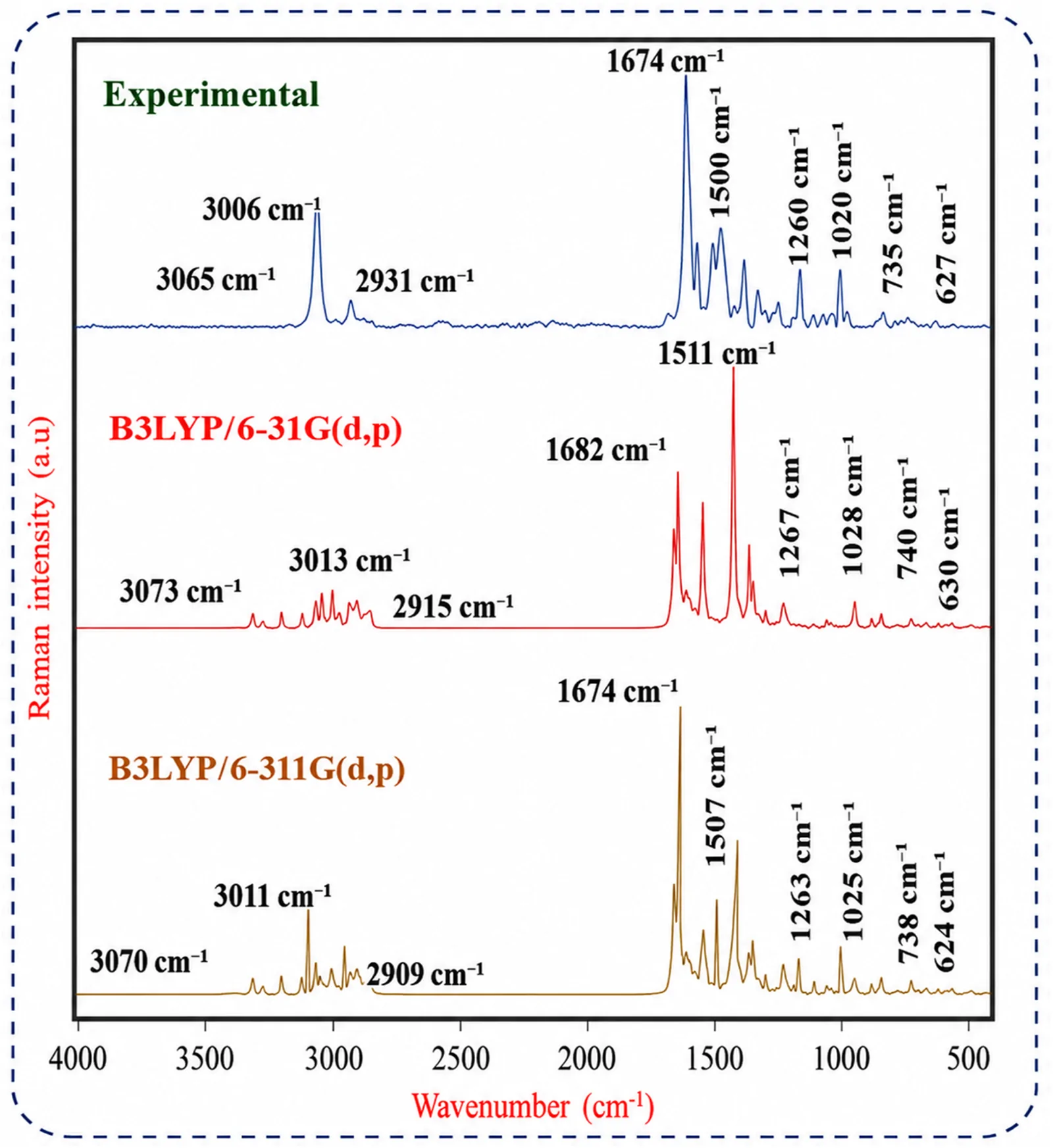
Experimental and calculated FT-Raman spectra of NHDIPSA.

The foundation of vibrating spectroscopy is a radiation-sensitive technique that relies on regular variations in polarizabilities (Raman) or dipole moments (IR) brought from vibrations of either atoms or molecules. The spectroscopic vibrating allocations in the basis groups 6-31G(d,p) and 6-311G(d,p) are counted. Specific Raman intensities, infrared intensities, and group frequencies.^28,29^ NHDIPSA is composed of 70 atoms with a total of 204 vibrations, of which 75 are stretching vibrations (*ν*), 65 are in-plane (*δ*), 25 are out-of-plane (*γ*) bending, and 39 torsion (*τ*) oscillations. The computed and observed spectra of FT-IR and FT-Raman are shown in Fig. 6 and 7. The normally occurring vibrational modes of NHDIPSA are detailed in Table S3, which also includes information on their associated vibrational assignments, frequencies (both theoretical and observed), and more. Eight distinct functional groups comprise NHDIPSA, and they are as follows: the vibrations include C–C (methyl), C–H (aromatic rings), C–O (phenolic), imidazole ring, C–N (aromatic amines/imidazole nitrogen), CH_3_ (methyl), O–H (phenol), and S–O (sulfonyl). The derived and observed wave numbers, as well as the distribution of potential energy. In order to do the vibrational analysis of the molecule, Table S4 provides a comprehensive reference list of all 349 internal coordinates. The key or legend provides an explicit definition of each motion, which is classified as stretching (*v*), bending (*δ*), torsion (*τ*), by outlining the precise order of atoms involved in that movement. For example, *v*1 is the N63–H64 stretch. The potential energy distribution (PED) may be understood by computational chemists by referring to this exhaustive set of definitions, which properly associates the computed percentages of vibrational frequencies with particular, chemically relevant bond stretches, angle bends, and dihedral twists.

#### 3.6.1 C–C vibrations

The stretching C–C oscillations have been reported between 1650 to 1200 cm^−1^ by Shamugapriya *et al.*^30^ The present investigation reveals methyl C–C *v* oscillations in the 1680–1299 cm^−1^ region. Very strong peaks at 1588 and 1403 cm^−1^ in the Fourier transform infrared (FT-IR) spectrum and the FT-Raman spectra at 1679 and 1605 cm^−1^. The vibrations at 493 and 230 cm^−1^, as well as 751, 447, and 128 cm^−1^, are detected. The distribution of maximal potential energy is 76%. The *δ* and *γ* bending vibrations of the benzene ring are generally observed at wavenumbers below 1000 cm^−1^.^31^ These modes are affected by substituents and other modes; hence, they are not pure in and of themselves. The aromatic ring C–C stretching vibrations in the NHDIPSA molecule are tightly coupled with other in-plane movements and span the 1669–1340 cm^−1^ spectral range. The experimental observation of a leading C–C stretching mode at 1669 cm^−1^ is in agreement with the B3LYP/6-311G(d,p) computed value of 1669, which has a Potential Energy Distribution (PED) of 38%. The FT-IR spectrum reveals another distinctive ring stretching vibration at 1585 cm^−1^, which shows a strong agreement with the theoretical prediction. At 653 cm^−1^ in the lower frequency region, the in-plane ring bending is clearly visible, which is in agreement with the 653 cm^−1^ calculated value at 68% PED. On the other hand, below 500 cm^−1^, the out-of-plane ring torsions are identified, which represent the non-planar twisting of the complex five-ring system. These skeletal vibrations define the structural stiffness and the extended conjugation of the molecule.

#### 3.6.2 C–H vibrations

It is common for phenyl groups to be the most prominent sites of stretching vibrations of C–H. The literature review revealed that the C–H stretch vibrations occur between 3100 and 3000 cm^−1^. Identified aromatic C–H stretching vibrations occur in the high-frequency range of 3193 cm^−1^ to 2884 cm^−1^. The Potential Energy Distribution (PED) purity of these modes is quite high, usually between 80% and 99%. Particularly noticeable are prominent bands at 3193 cm^−1^ when 95% PED, 3114 cm^−1^ when 99% PED, and 3065 cm^−1^ when 91% PED are present. The modes of in-plane bending are dispersed over the 1231–1017 cm^−1^ array. These vibrations are generally coupled with aromatic C–C stretch. Two distinct bands are observed at 1231 cm^−1^ (73% PED) and 1224 cm^−1^ (76% PED). In the region of frequencies from 716 cm^−1^ to 619 cm^−1^, there are out-of-plane bending vibrations that are often combined with torsional movements. The percentages of PED contributions in these modalities vary between 34% and 59%. So, assuring the strong agreement between the two. The assignments are further backed up by Potential Energy Distribution (PED) values up to 99%, which show that these stretching modes have very high group purity.

#### 3.6.3 O–H vibrations

For the vibratory stretch of O–H, the predicted wavelength is 3380 ± 200 cm^−1^ as stated in reference. The infrared emission bands and density-functional theory (DFT) both demonstrated the stretching vibration for O–H at 3655 cm^−1^ and 3661 cm^−1^, respectively, as reported by Benzon *et al.*^32^ the infrared spectra showed the vibratory stretch of the O–H bonds at 3741 cm^−1^ as reported by Goutam Brahmachari *et al.*^33^ The vibratory stretch of O–H was detected in the infrared spectrum at 3336 cm^−1^ as reported by Subashini *et al.*^34^ As shown in this study, the O–H vibrations are identified in Fourier transform infrared (FT-IR) at 3267 cm^−1^, but they are not detectable in Fourier transform Raman (FT-Raman). The wavenumbers *v* = 3271 and *γ* = 580 cm^−1^ were obtained by the B3LYP/6-311G(d,p) method and *v* = 3275 and *γ* = 582 cm^−1^ by the B3LYP/6-31G(d,p) method, respectively. Potential energy distribution at its maximum is 98%. Experimental observations of O–H stretching vibrations are in the 3300–3740 cm^−1^ range, but theoretical calculations using B3LYP techniques indicate values in the 3310–3312 cm^−1^ range. The good agreement shows the reliability of both practical and theoretical results.

#### 3.6.4 C–O vibrations

The C–O stretching mode is usually expected to appear in the range 1850–1550 cm^−1^.^35,36^ C–O stretching modes were observed at 1032 cm^−1^ (IR), 1188 cm^−1^, 1035 cm^−1^, 955 cm^−1^ and 892 cm^−1^ (DFT).^37^ In their study the carbon–oxygen vibrations of stretching in the IR spectrum are 1617 cm^−1^ and in the Raman spectra are 1614 cm^−1^ and the theoretical value is 1649 cm^−1^.^38^ The stretching modes of C–O are detected at 1786 cm^−1^, 1603 cm^−1^, and 1027 cm^−1^ in the Fourier transform (IR) spectra, and at 1726 cm^−1^ and 1629 cm^−1^ in the Fourier transform (FT-Raman) spectra. The stretching modes of C–O were identified by Xavier *et al.*^39^ at 1260 cm^−1^, 1250 cm^−1^, and 1240 cm^−1^ in the FT-IR spectra, and at 1250 cm^−1^ and 1240 cm^−1^ in the FT-Raman spectra.

The present study reveals that the C–O stretching vibration of the hydroxy group is often considered as a mode mixed with C–C stretching. It is observed at 1306 cm^−1^ in the FT-IR spectra and at 1313 cm^−1^ in the theoretical calculations. The presence of stretching vibration of C=O group of carbonyl is seen as a medium-strong band at 1675 cm^−1^ (FT-IR) and weak band at 1674 cm^−1^ (FT-Raman) with Potential Energy Distribution (PED) of 57%. The C–O–C group's in-plane bending is specified by the internal coordinate involving the C28–O38–C29 atoms. The carbonyl group's (C=O) out-of-plane motion is given by the atomic coordinates O66–C65–N63–C67. There are further contributions to C–O stretching at 1500 cm^−1^ and 1317 cm^−1^, which are often accompanied by N–C and C–C stretching movements. The FT-IR spectrum shows the stretching vibration of the hydroxyl group (C–O) at 1306 cm^−1^, which is in agreement with the calculated value of 1313 cm^−1^. Theoretical calculation of the vibrational spectra was done by density functional theory (DFT) and supported the assignment of the vibrational modes in FT-IR and FT-Raman spectra.

#### 3.6.5 C–S vibrations

Band assignments resulting from C–S vibrations of stretching in various substances are notoriously challenging. Both aromatic and aliphatic sulfides have a low to moderate spectrum because of the vibration of the stretched C–S bond in the 1110–380 cm^−1^ region. The pattern of C–S stretch modes was seen at 729 cm^−1^ in the Raman spectra and 728 cm^−1^ in the IR spectrum, as reported by Aswathy *et al.*,^40^ and Kuruvilla *et al.*^41^ found the stretching modes of C–S in the infrared spectrum at 714 cm^−1^. Although El-Azabet *et al.*^42^ theoretically calculated the C–S modes of stretching at 797 and 635 cm^−1^, in the Raman spectrum, they are reported at 632 cm^−1^. The band was detected in the current investigation at 1073 ms cm^−1^ in the FT-IR and 1061 ms cm^−1^ in FT Raman. The C–S stretching vibrations are notably characterized by the bands detected at 802 cm^−1^ (FT-IR) and 735 cm^−1^ (FT-Raman). The theoretical B3LYP/6-311G(d,p) calculations for these stretching modes are 809 cm^−1^ and 738 cm^−1^, respectively. The Potential Energy Distribution (PED) for the C–S stretch at 802 cm^−1^ is 64%, which means that this is the main motion for that mode. In-plane bending vibrations that include the sulphur atom, such as the S–C–C and C–C–S angles, are found in the lower frequency range. Skeletal torsions that include the C–S bond, such as the C–C–C–S torsion, are in the lowest frequency range of the spectrum, which is 5 cm^−1^. These numbers show that the experimental observations and the DFT calculations for the sulfur–carbon bonds are quite similar. The PED values for these modes (47% to 64%) show that they are mostly C–S stretches, but they also show some connection with skeletal torsions and ring deformations.

#### 3.6.6 CH_3_ vibrations

The methyl group in the NHDIPSA molecule shows stretching, deformation and torsional modes of vibrations which are in very good agreement with experimental and theoretical data. The asymmetric stretching vibrations are determined to be 2872 and 2865, with Potential Energy Distribution (PED) contributions of 88% and 87%, respectively 1. The symmetric stretching vibration is seen in the FT-IR spectrum as a medium-intensity band at 2851 cm^−1^, which is very near to the estimated value of 2855 cm^−1^ and a PED of 91% of 2. The methyl scissoring motion is found at 1435 cm^−1^ with a high PED of 92% of 3 for the deformation modes. In the low-frequency range, the methyl torsion adds to complicated skeletal modes. This happens below 150 cm^−1^, it is feasible to achieve an energy distribution of up to 98%.^43^ Although hyperconjugation is a well-established chemical theory for methyl group frequency shifts in aromatic systems, the presented data demonstrate that the assignments are correct by means of Potential Energy Distribution (PED) and the high agreement between estimated and measured values.

#### 3.6.7 N–H vibrations

There are clear spectrum fingerprints in the high- and low-frequency bands for the N–H group vibrations in the NHDIPSA molecule, and the group purity is very high overall. In the FT-IR spectrum, the medium intensity band at 3306 cm^−1^ represents the N–H stretching vibration. This band fits very well with the theoretical B3LYP/6-311G(d,p) prediction of 3310 cm^−1^ and displays a 100% Potential Energy Distribution (PED). The mode at 1675 cm^−1^, which is associated with the carbonyl C=O stretching vibration, is greatly influenced by the in-plane bending motion. The bending modes occur, as shown in Table S3. In addition, mode 140 is attributed to the N–H out-of-plane bending, which is computed at 569 cm^−1^ and has a PED of 62%. The results enable the highly localized N–H vibrations to be used as signatures for the environment of the secondary amine in the molecular framework. These high frequency assignments together give a very reliable correlation between the FT-IR data and DFT computations for the polar functional groups.

#### 3.6.8 SO_2_ vibrations

SO_2_ symmetrical and asymmetrical stretching vibrations were identified in the 800–720 cm^−1^ and 900–835 cm^−1^ bands. The results obtained from the levels at B3LYP/6-31G(d,p) and 6-311G(d,p) sets of bases, which show bands at *υ* = 840 cm^−1^ and 835 cm^−1^, are ascribed to the symmetrical and asymmetrical of SO_2_ vibrations of stretching. The SO_2_ scissoring vibrations were detected in the 560 ± 40 cm^−1^ range. The computed spectrum was visible at *δ*_sciss_ = 292 cm^−1^ and *δ*_sciss_ = 289 cm^−1^. The oscillation occurs at approximately 350 cm^−1^. The spectrum noticed at *δ* = 270 cm^−1^ and *δ* = 269 cm^−1^ were computed of the title compound. The SO_2_ rocking vibrations seen within the range of (530 ± 50 cm^−1^). The computed spectrum formed at *δ*_rock_ = 209 cm^−1^ and *δ*_rock_ = 206 cm^−1^. The positioning of wagging is appeared at (500 ± 55 cm^−1^). In the NHDIPSA spectrum, the vibrations of the sulfonyl group stand out as very strong and distinctive. The FT-IR spectrum reveals the presence of the asymmetric stretching vibration at 1328 cm^−1^, which is well correlated with the estimated value of 1337 cm^−1^ (84% PED). In FT-IR and FT-Raman, the symmetric stretching vibration is detected as a very strong band at 1161 and 1162 cm^−1^, respectively, which is in perfect agreement with the theoretical prediction of 1165 cm^−1^ (85% PED). Scissoring at 720 cm^−1^ (68% PED), wagging at 399 cm^−1^ (63% PED), and rocking at 206 cm^−1^ (63% PED) are the three equally well-defined deformation modes. The sulfonyl group vibrations are considerably isolated from the surrounding molecular framework, as shown by the high PED values for these modes. Confirming the precision of the B3LYP/6-311G(d,p) basis set in modelling the sulphur–oxygen double bond character, the experimental and theoretical values for the sulfonyl group show minor variance, generally about 10 cm^−1^. The very high PED values (60%) show that these modes are confined and unaffected by the vibrations of the aromatic rings that are connected.

#### 3.6.9 Ring vibrations

The skeletal vibrations of NHDIPSA arise from the intricate five-ring structure. The region 1669–1340 cm^−1^ is crowded with aromatic C–C stretching vibrations, normally accompanied by C–H in-plane bending. A prominent ring internal in-plane bending mode (CCC) is found at 653 cm^−1^ (68% PED). The bands at 1263 cm^−1^ and 1260 cm^−1^ (68–70% PED) are attributed to the C–N stretching modes that connect the rings and the functional groups. Below 500 cm^−1^, the low frequency region is dominated by ring and inter-ring torsions (CCCC). These torsions indicate the non-planar twisting of the rings in relation to one another. It goes up to 5 cm^−1^ (60% PED) and ends with the last skeletal torsion (CCCS) which is the lowest energy normal mode of the system. The good agreement of these modes demonstrates the correctness of the force constants and molecular geometry calculated for the fused ring system.

#### 3.6.10 Validating the vibrational assignments

The exact correlation between experimental results and B3LYP/6-311G(d,p) theoretical computations over all 204 normal modes validates the vibrational study of the NHDIPSA molecule. The N–H and O–H stretching vibrations (3306–3267 cm^−1^) and other important polar groups exhibit very accurate and dependable assignments with Potential Energy Distribution (PED) values ranging from 98–100%. The carbonyl (C=O) stretches at 1675 cm^−1^ and the sulfonyl (SO_2_) asymmetric and symmetric stretches at 1328 cm^−1^ and 1161 cm^−1^, which are all in good agreement with DFT predictions, anchor the mid-frequency region. Accurately predicted C–S stretching (802 cm^−1^) and ring in-plane bending (653 cm^−1^) in the fingerprint area (below 1000 cm^−1^) validate the structure. The good agreement between the predicted frequencies and the observed intensities confirms the correctness of the electronic environment and the calculated structure of the NHDIPSA molecule. This agreement in all tests also attests to the reliability of the experimental assignments as well as to the accuracy of the theoretical methods used.

### 3.7 Frontier molecular orbitals

**Fig. 8:**
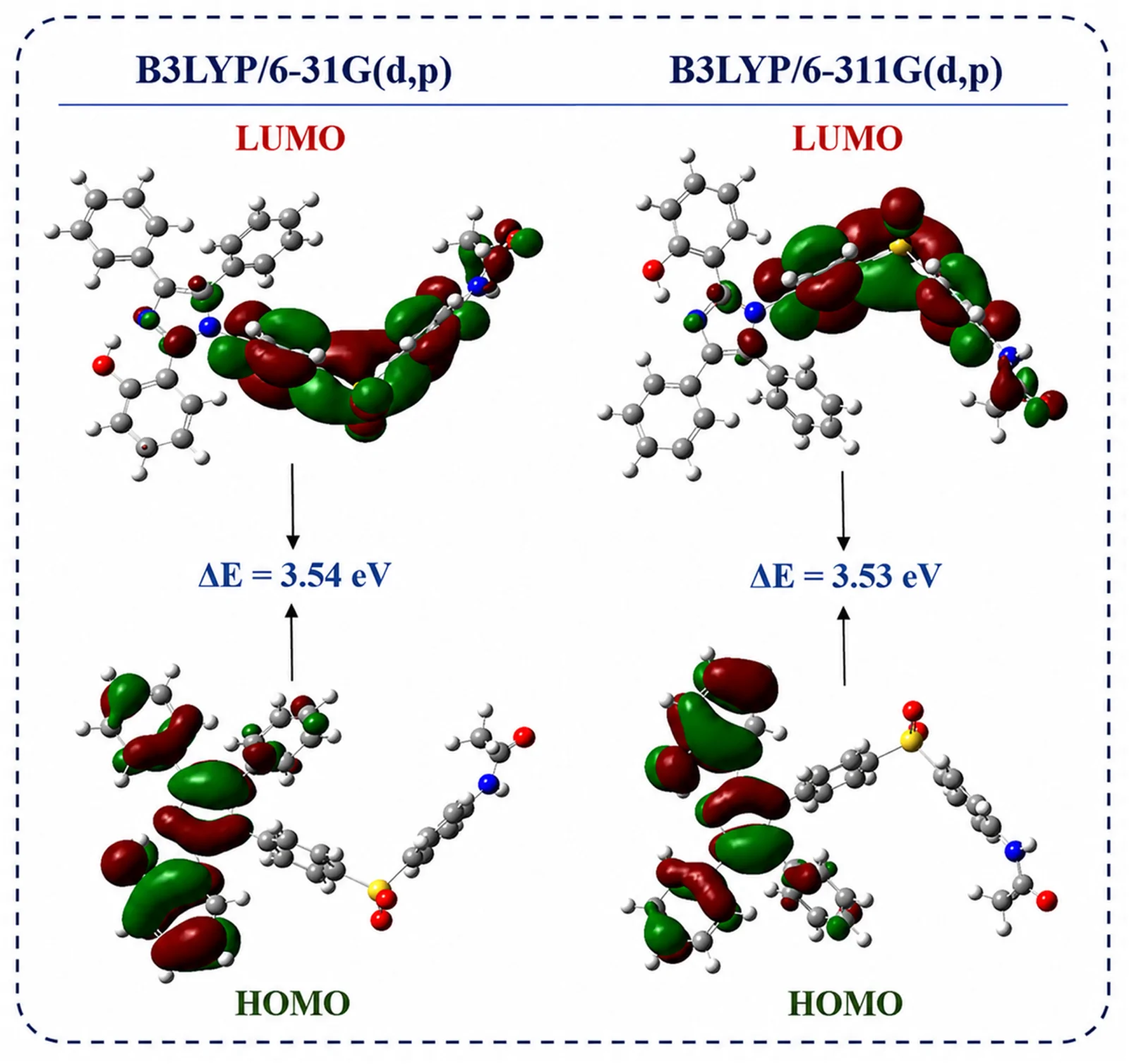
HOMO–LUMO diagrams of NHDIPSA.

By calculating the difference between the energies of (HOMO) and (LUMO),^44^ the molecular properties such as chemical stability, kinetic stability and optical polarizability can be obtained thru simple method. Frontier molecular orbital theory is well known for its applicability to predict the qualitative aspects of many chemical processes.^45–47^ Frontier molecular orbital theory largely determines the optical and electrical properties of a substance. The HOMO–LUMO diagrams of NHDIPSA are given in Fig. 8. In a HOMO state, an electron may be given or taken at the same time, but in a LUMO state, an electron can only be taken. Quantification of chemical reactivity is done by calculating the electrophilicity index, chemical hardness, ionization capacity, chemical softness, and the attraction for electrons based on the energy levels of the HOMO and LUMO orbitals.^48^

The global reactivity descriptors were calculated based on the energies of the Highest Occupied Molecular Orbital (*E*_HOMO_) and the Lowest Unoccupied Molecular Orbital (*E*_LUMO_). According to Koopmans' theorem,^49^ the ionization potential (*I*) and electron affinity (*A*) are defined as:

2 *I* = −*E*_HOMO_

3 *A* = −*E*_LUMO_

Using these values, the electronegativity (*χ*), chemical hardness (*η*), chemical softness (*S*), and electrophilicity index (*ω*) were determined using the following equations:

4 Electronegativity: *χ* = *I* + *A*/2

5 Chemical hardness: *η* = *I* − *A*/2

6 Global softness: *S* = 1/*η*

7 Electrophilicity index: *ω* = *χ*^2^/2*η*

These descriptors provide a quantitative measure of the stability and reactivity of the NHDIPSA molecule.

It is an important factor in the energy gap and the global reactivity descriptors (Δ*E*) between chemical orbital spaces.

8 Δ*E* = *E*_HOMO_ − *E*_LUMO_

The frontier molecular orbital (FMO) characteristics of NHDIPSA were calculated using B3LYP/6-31G(d,p) and B3LYP/6-311G(d,p) basis sets in the framework of density functional theory. The HOMO energies were calculated as −5.54 eV and −5.80 eV and the LUMO energies were −2.00 eV and −2.27 eV for the respective basis sets. The HOMO–LUMO energy gaps obtained were 3.54 eV and 3.53 eV, respectively, for both cases, indicating that the electronic behaviour is consistent at both levels of theory. A moderate energy gap suggests that the molecule has reasonable kinetic stability and a balanced degree of chemical reactivity. Other global reactivity descriptors also support this finding. The electronegativity values were obtained as 3.77 and 4.03 eV and the values of global hardness were 1.77 and 1.76 eV for the two basis sets respectively. The corresponding electrophilicity indices were calculated as 4.01 and 4.60 eV, which indicates that NHDIPSA has appreciable electrophilic character with moderate reactivity. The global softness values (∼0.28 eV^−1^) also suggest a good capability to undergo charge transfer interactions. The very similar values of the electronic descriptors calculated with the two basis sets indicate a good convergence to the basis set limit and, consequently, reliability of the computational methodology. Table 2 also shows the HOMO–LUMO energy gap and other reactivity parameters. The HOMO–LUMO energy gap is not a direct predictor of biological or docking performance but rather an indicator of molecular stability and trends in reactivity.

**Table 2:** NHDIPSA's global electronegativity, HOMO, LUMO, hardness, and softness, as well as its global electrophilicity index

Parameter	Theoretical	
	B3LYP/6-31G(d,p)	B3LYP/6-311G(d,p)
*E*_HOMO_ (eV)	−5.54	−5.80
*E*_LUMO_ (eV)	−2.00	−2.27
Δ*E* (*E*_HOMO_ − *E*_LUMO_) gap (eV)	3.54	3.53
Electronegativity (*χ*)	3.77	4.03
Global hardness (*η*)	1.77	1.76
Global softness (*σ*)	0.282	0.284
Electrophilicity index (*ω*)	4.01	4.60

### 3.8 Molecular electrostatic potential (MEP)

**Fig. 9:**
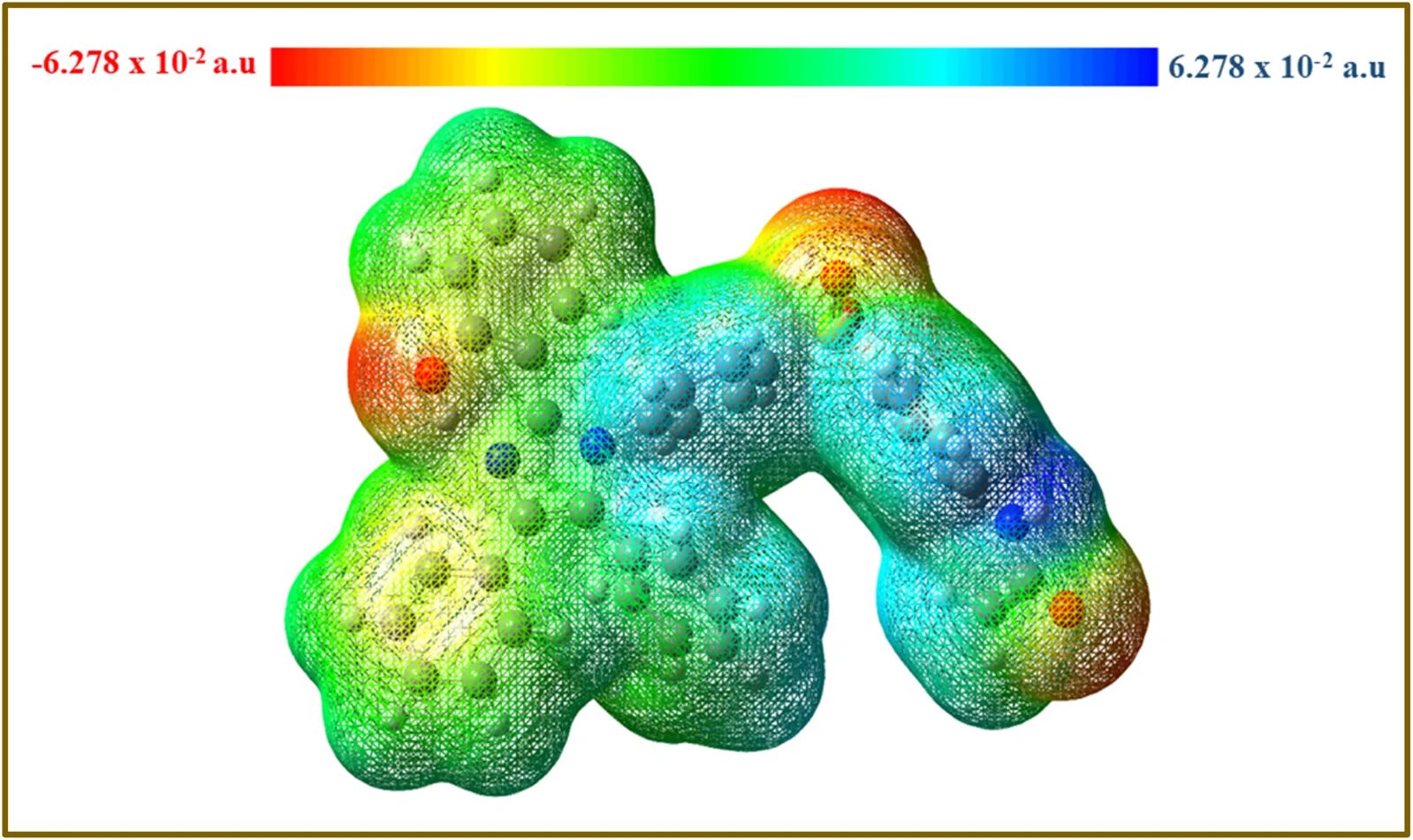
Molecular electrostatic potential for B3LYP/6-311G(d,p) of NHDIPSA.

The Molecular Electrostatic Potential (MEP) is one of the useful tools to study the distribution of electric charges on molecules. The electrostatic field can be used to predict where an attack might occur by identifying reactive regions in a molecule.^50–52^Fig. 9 shows the process of creating a rainbow-colored map of the molecule's MEP surface graphs. The colour coding within this map provides a transparent representation of the allocation of charges, graphically illustrating them.

Using a color coding, the mapping reveal's locations with strong negative charge (red), high positive charge (blue), electron rich regions (yellow), zero potential zones (green), and intermediate charge levels (other colours).^53,54^ The color scale, ranging from red through yellow, green, and blue, represents increasing electrostatic potential across the molecular surface, from the most negative (red) to the most positive (blue) regions. The molecular electrostatic potential delineation of the molecule under discussion in this instance begins with red (−6.278 × 10^−2^ a.u.) and concludes with dark blue (6.278 × 10^−2^ a.u.), indicating changes in the electrostatic potential throughout the molecule's surface. Electron delocalization is expected to occur predominantly around the oxygen, sulphur, and nitrogen atoms, which correspond to the red (negative) and blue (positive) regions of the MEP surface.

The nitrogen atoms of imidazole ring are located in a region of positive electrostatic potential. The sulfonyl (SO_2_) group and the oxygen of the carbonyl (C=O) of the amide group are in the negative electrostatic potential region in accordance with their electrophilic and electron-withdrawing nature. This zone of positive also contributes to the max electrostatic potential because there are nucleophilic hydrogen and nitrogen atoms located in the area. The MEP map displays the chemical reactivity and chemical active position of atoms. The MEP calculation shows the electron-rich and electron-deficient regions which may take part in the intermolecular interactions and complements the qualitative observations from docking.

The electron-rich regions centered on the carbonyl oxygen, sulfonyl oxygens, and imidazole nitrogen, together with the electron-deficient amide N–H and hydroxyl O–H, correspond to the same functional groups that form hydrogen bonds with active-site residues in the docking poses described in Section 3.11. This agreement is descriptive: it shows internal consistency between the ligand's calculated reactivity and its docking behavior, and it does not by itself establish or predict biological potency.

### 3.9 Topological analysis

Topological data analysis is crucial as it enables the extraction of meaningful insights from complex data by emphasizing the underlying shape and structure, even in the context of high-dimensional datasets. This analysis utilizes several parameters, including Localised Orbital Locator (LOL), Electron Localization Function (ELF), and Non-Covalent Interaction (NCI) – RDG (Reduced Density Gradient).^55^

#### 3.9.1 Electron localization function (ELF)

**Fig. 10:**
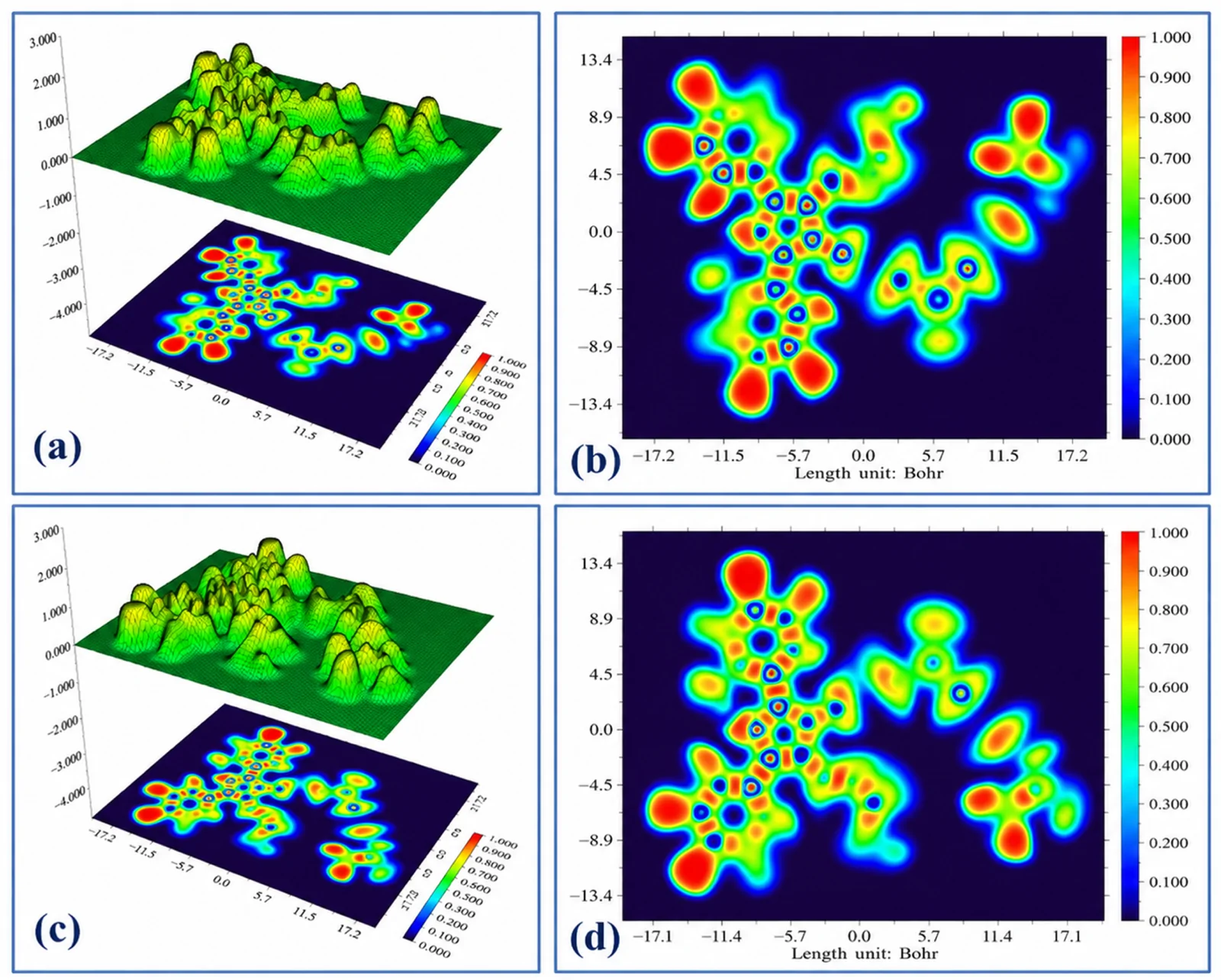
(a) 3D-highlighted surface of projection and (b) Electron Localization Function (ELF) colour filled map for B3LYP/6-31G(d,p); (c) 3D-highlighted surface of projection and (d) Electron Localization Function (ELF) colour filled map for B3LYP/6-311G(d,p) values of NHDIPSA.

To better understand what makes molecules stable and reactive, topological research is a wealth of information.^56^ When expressed as an activity of electron pairs each unit of area, ELF is determined by using orbital gradients. An important tool in computational chemistry, the Electron Localization Function (ELF) measures *via* the electron's localization near the nuclei of an atoms. It helps in the study of chemical bond by separating them into metallic, covalent, and ionic bonds and by locating areas with a high electron density, which are probable sites of reactions. We can learn more about chemical bonding and reactivity with the use of this function, which sheds light on the electronic structures of molecules and materials. When looking for opposing spin-pairs or single electrons, Pauli repulsion, which is generated by an excess of kinetic energy population density, is the way to go. Strong Pauli attraction, indicates an ELF value around to 1, while strong Pauli repulsion, indicates an ELF value close to 0. The Multiwfn software was employed to generate the colour-highlighted surface-filled projection map of the NHDIPSA molecules for B3LYP/6-31G values and the shaded surface relief map of ELF for 6-311G(d,p) values Fig. 10. *τ*(*r*) is a typical way to represent ELF. A blue-to-red hue gradient denotes a range of scale from 0.000 to 1.000 on the ELF map. The Bohr magnitudes for these zones in set 1 are −17.2 to 17.2, whereas in B3LYP/6-311G(d,p) they are −17.1 to 17.1. Red areas surrounding hydrogen atoms represent areas of high ELF values, blue areas represent bonded and nonbonding electrons, and red, blue, oxygen, sulfur and nitrogen atoms are expected to show electron delocalization.

#### 3.9.2 Localized orbital locator (LOL)

**Fig. 11:**
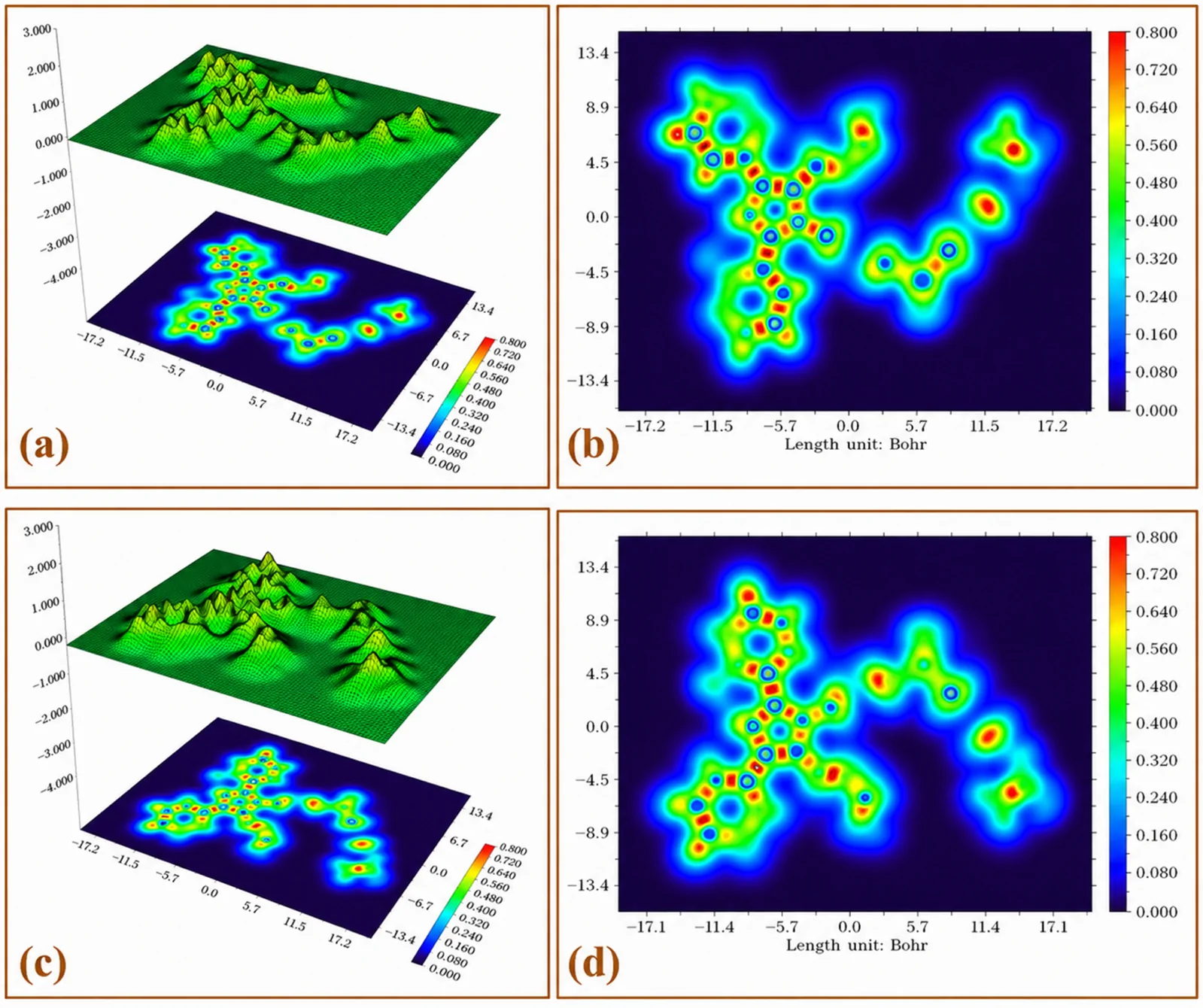
(a) Projection of 3D shaded surface and (b) localized orbital locator (LOL) color filled map for B3LYP/6-31G(d,p); (c) 3D shaded surface of projection and (d) colour-filled map of the localized orbital locator (LOL) for B3LYP/6-311G(d,p) value of NHDIPSA.

The Localized Orbital Locator (LOL) is an important device used for investigating a molecule's internal chemical bond connections. According to LOL, the orbital gradient is responsible for the largest amount of orbital localization and overlap.^57^ The LOL function calculates numerical closeness of localized orbitals to individual nuclei or groups of nuclei by analyzing their position within the molecular structure. The letter “*r*” stands for LOL. By drawing attention to places with strong or weak peak regions, this relief map shows the electron distribution around specific atoms. Many researchers use the LOL to study molecular arrangement, responsiveness, and connections. Methods such as ELF analyze the distribution of energy resulting from motion to conduct investigations. In the two-dimensional model, the blue circle denotes a sparsely populated area between the internal and external electron shells, while the dark red-the color orange region shows heavy localization of electrons. The measured LOL values vary from 0 (blue) to 0.8 (red) on the colour scale that goes from 0.000 to 0.800. On this scale, red represents the highest point and blue the lowest. The red region occupies from 0.800 to 0.640. The yellow region occupies from 0.640 to 0.560 the green from 0.560 to 0.320 and the blue region occupies from 0.320 to 0.000 for both basics set values. The values span from −17.2 to 17.2 for B3LYP/6-31G(d,p) and −17.1 to 17.1 for B3LYP/6-311G(d,p) values Fig. 11. Atoms of nitrogen, carbon and oxygen are coloured blue to represent their electron distribution, whereas atoms of hydrogen are coloured red to represent their firmly bonded electrons.

#### 3.9.3 Non-covalent interactions (NCI)

**Fig. 12:**
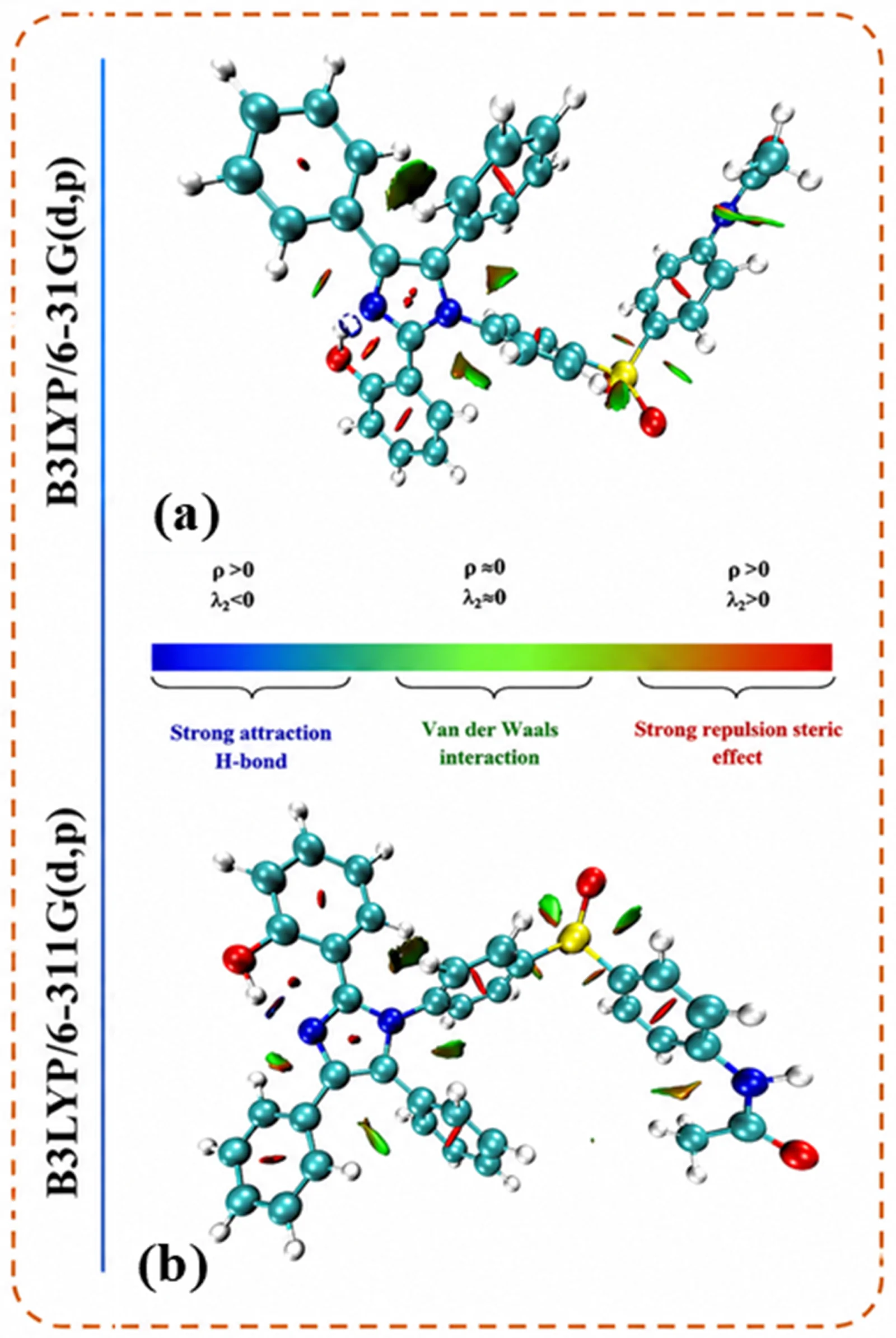
(a) 3D-RDG surface visualization for (B3LYP/6-31G(d,p)); (b) 3D-RDG surface visualization for (B3LYP/6-311G(d,p)) of NHDIPSA.

One topological approach to discovering NCIs, or bonds composed of hydrogen, van der Waals and steric effects actions, is RDG paradigm. The Reduced Density Gradient (RDG) technique is a helpful device for studying the molecular structures, especially for detect ineffective intermolecular forces like as interacting with van der Waals mechanism and hydrogen bonding, in conjunction with looking at different chemical reactions and steric effects inside molecules.^58^ Weak interaction zones are located in regions with low electron density and modest density gradients, while high-density gradients suggest the existence of strong interaction zones linked to chemical reactivity in regions with high electron densities. In Fig. 12, the relationship between *ρ*(*r*), the electron density at position *r*, and the electron density's second Hessian eigenvalue, which is represented by as sign (*µ*^2^) *ρ*, plotted against *ρ* and the latter is a dimensionless quantity which is defined mathematically by eqn (9):9
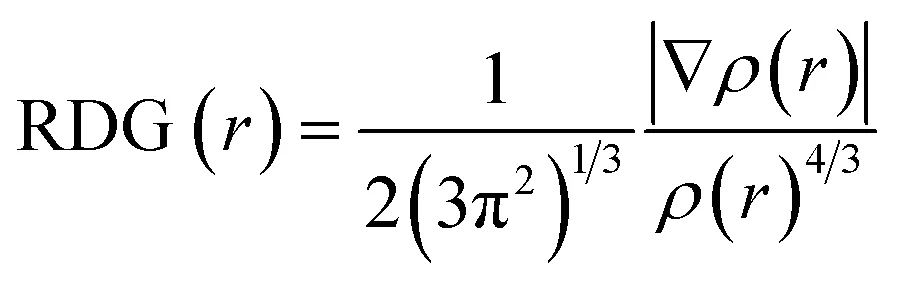


Fig. 12(a and b) shows the 3D RDG surface visualization of N2HIA for B3LYP/6-31G(d,p) and 6-311G(d,p) values, generated by the tools Multiwfn and VMD. In the 3D RDG iso surfaces, different kinds of interactions are shown by different colours. Hydrogen bonds are represented by blue, interactions of van der Waals force by green, and strong repulsive forces, often called steric effects in rings and cages, by red. Negative values of (*λ*^2^) *ρ* indicate high attractions, and positive values denote strong repulsion, correspondingly, and the value of van der Waals interactions are close to zero.

Based on the point scattering diagram, which shows levels ranging between 0.020 to 0.010 a.u., the red fragmented parts in the aromatic ring center are likely due to steric effects. Similar to the decreased density gradient shown in the low-density region between −0.025 and −0.035 a.u., the blue region represents strongly engaging interactions. As demonstrated in the RDG curve between 0.000 and −0.015 a.u., the area of green flaky patches material suggests that the methoxy group has weak non-covalent interactions. Both values in the set provide identical readings. Neither of these measurements deviates from the fundamental set values. Readings for both of the fundamental set values are identical for the chemical reactions in methoxy group, as seen in the RDG curve between 0.000 and −0.015 a.u.

### 3.10 Ramachandran plot for various docking proteins

**Fig. 13:**
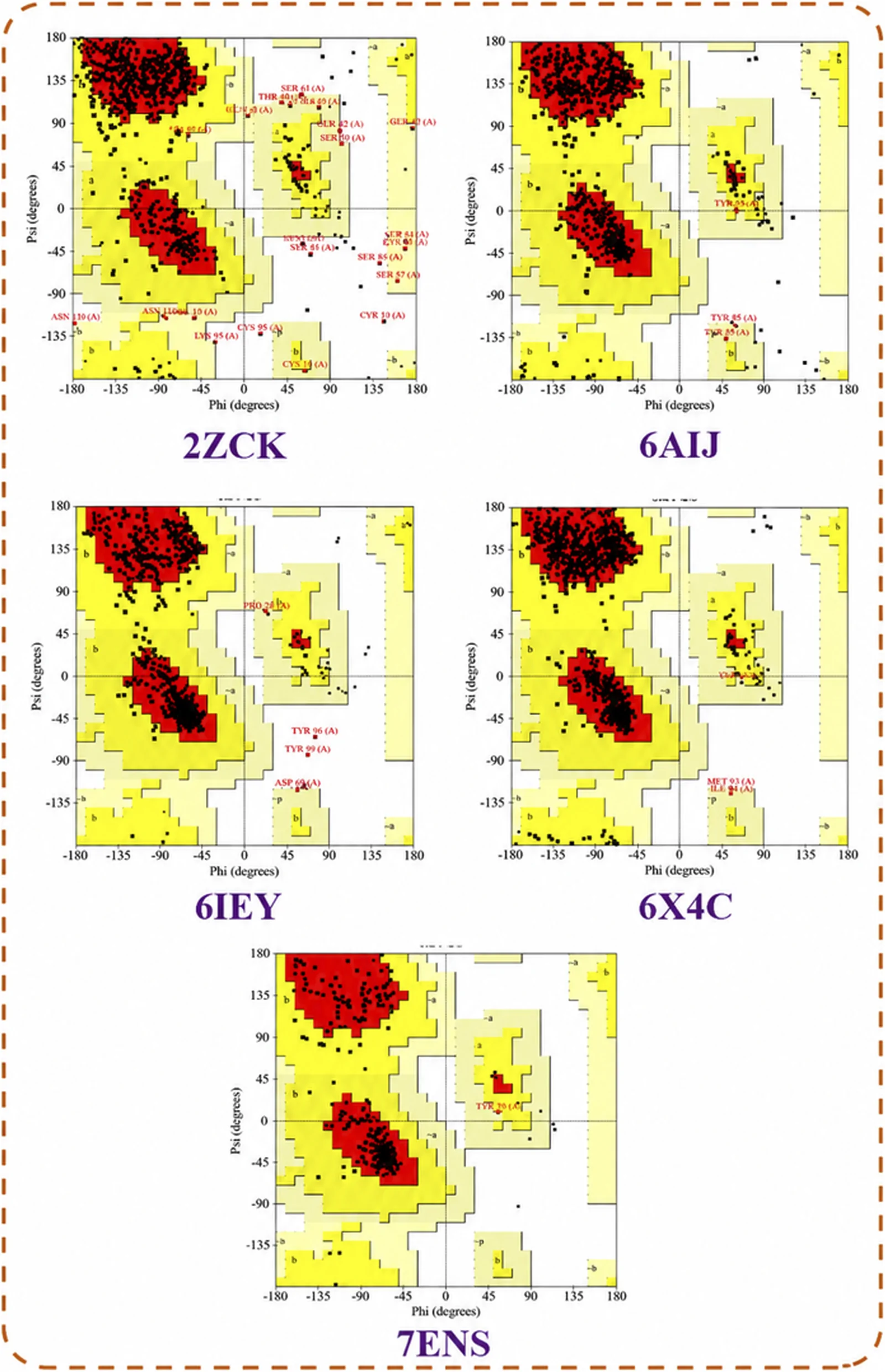
Ramachandran plots of the five target proteins (2ZCK, 6AIJ, 6IEY, 6X4C, and 7ENS) used in the molecular docking study of NHDIPSA.

The Ramachandran plot was useful in confirming the structure of the protein. The density of points of data derived from the library of strongly improved structural proteins makes the limits of the plot very apparent. It provides a statistical representation of the distribution of amino acids. Since the protein's molecular structure is stable using the Ramachandran plot, it was selected for the docking research.^59^ Most of the residues are located in the red region and yellow areas, which are known as the permitted and core regions, respectively, as shown in Fig. 13. There are very few artifacts discovered in the off-limits regions. In Table 3, we can see that the results from the Ramachandran plot reveal that out of all the proteins 6X4C has the highest number of hydrogen-bonded proteins (948), followed by 2ZCK, 6AIJ, 6IEY, and 6X4C's hydrogen-bonded proteins (692, 687, 616 and 330, respectively). The allowed region of 7ENS has more residues than the other proteins. This proves that it is more stable now.

**Table 3:** Ramachandran plot statistics for the five target proteins (2ZCK, 6AIJ, 6IEY, 6X4C, and 7ENS) used in the molecular docking study of NHDIPSA

	2ZCK	6AIJ	6IEY	6X4C	7ENS
Ligand bound	Myristic acid	Ferulic acid	Cinnamic acid	Ibuprofen	Furosemide
Number of residues involved in hydrogen bonding (H-bonds)	692	687	616	948	330
Number of residues in the area that are most favoured regions	477	523	470	762	263
Number of residues in the extra areas allowed regions	91	58	44	53	25
Number of residues in the disallowed regions	7	0	3	1	0

### 3.11 Molecular docking

The binding affinity values are derived from the AutoDock Vina scoring function and represent relative binding compatibility with the docking model used; they are not indicative of pharmacological potency, which can only be determined by biochemical or cell-based assays. Molecular docking study molecular docking simulations were carried out using the AutoDock Vina 1.1.2 software tool. The search exhaustiveness was set to eight (8) to ensure adequate sampling of the ligand degrees of freedom within the grid-box. From the RCSB Protein Data Bank,^11^ we collected the crystal structures of the target proteins 6AIJ, 6IEY, 6X4C, and 7ENS. Before adding polar hydrogens and Kollman charges, the proteins were prepped by eliminating co-crystallized ligands and water molecules using Auto Dock Tools (ADT). The optimized structure of NHDIPSA ligand was converted to PDBQT format at B3LYP/6-311G(d,p) theoretical level and all rotatable bonds were set to flexible. Initially, a blind docking protocol was used to determine potential binding pockets, and then a site-specific docking protocol was used. The grid box was centered on the active site residues of each protein with a grid size of 126 × 126 × 126 Å and a grid spacing of 0.375 Å. We set the exhaustiveness option to 8 to ensure a complete search of the binding modes.

**Fig. 14:**
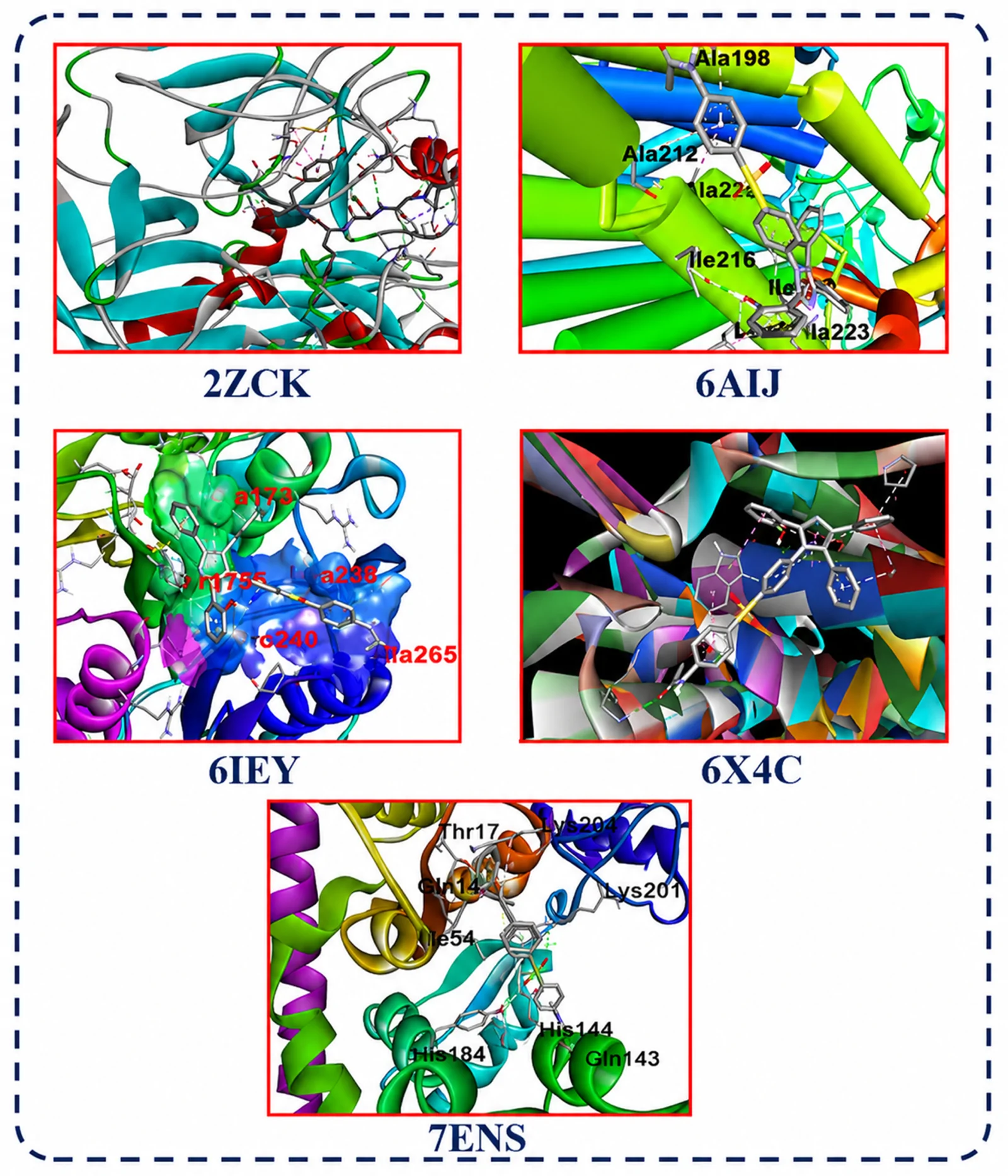
3D binding pose of NHDIPSA with studied proteins were performed using PyMOL and BIOVIA Discovery Studio Visualizer.

**Fig. 15:**
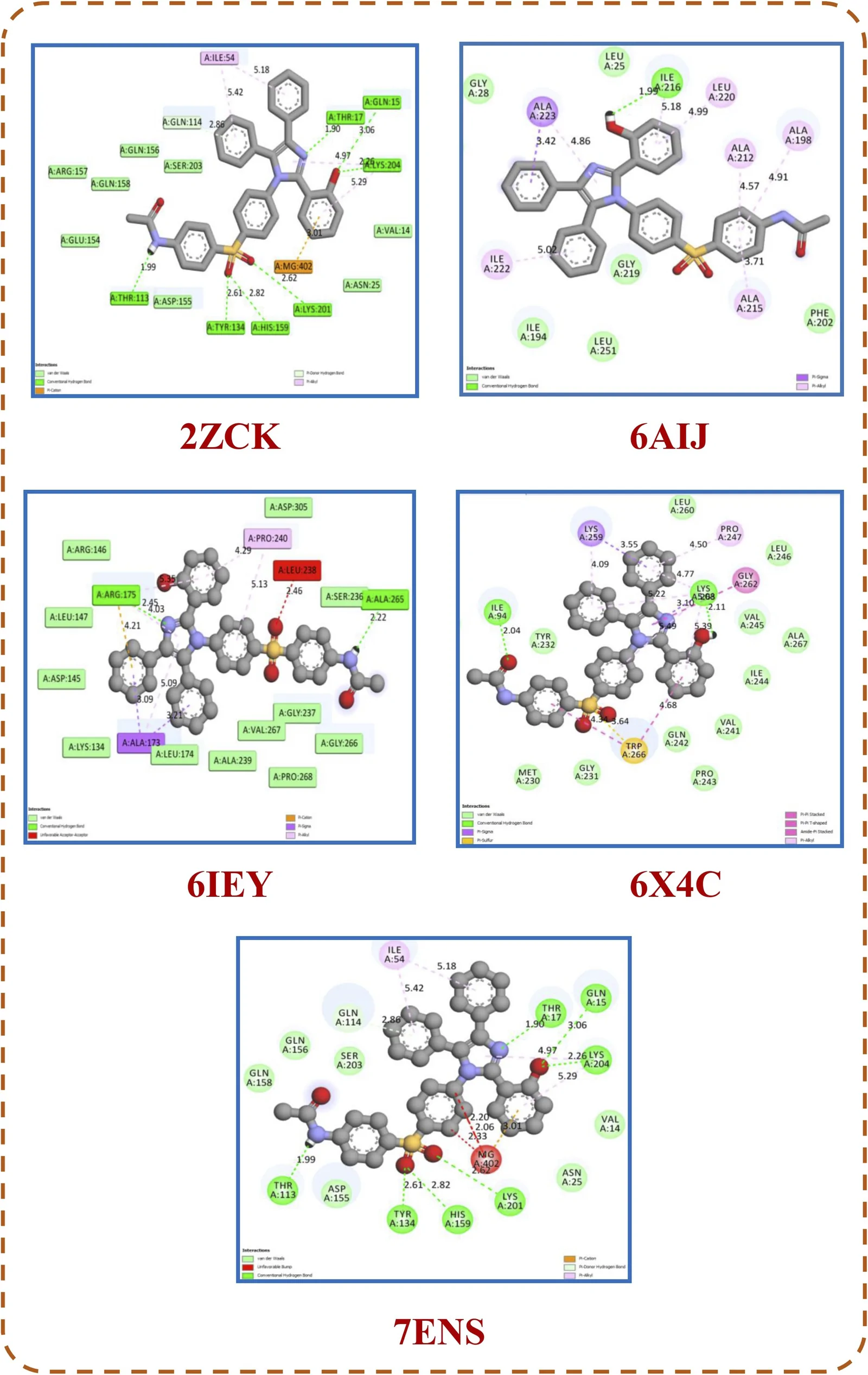
2D interaction of protein–ligand of NHDIPSA were performed using PyMOL and BIOVIA Discovery Studio Visualizer.

The simulation of the docking was performed using a flexible-ligand/rigid-protein model. To examine hydrogen bonding and hydrophobic interactions, the lowest binding affinity (kcal mol^−1^) was used to choose the best-posed conformation, which was then displayed using PyMOL and Discovery Studio Visualizer. Fig. 14 and 15 respectively, show the 3D and 2D binding posture and interaction of protein–ligands of NHDIPSA. Various types of interactions, such as hydrogen bonding, π–π stacking, hydrophobic interactions, and van der Waals interactions, occur in the docking complex.^60–63^ PASS (Prediction of Activity Spectra for Substances) studies indicate that NHDIPSA may possess antibacterial (*P*_a_ > 0.174), anti-obesity (*P*_a_ > 0.207), anti-infective (*P*_a_ > 0.242), (*P*_a_ > 0.239) Insulin inhibitor and anti-mycobacterial (*P*_a_ > 0.459) properties. According to the outcomes of the PASS analysis (Table 4), five unique proteins were tested for their interactions with ligands: 2ZCK, 6AIJ, 6IEY, 6X4C, and 7ENS. This study evaluated the computationally predicted antibacterial, anti-obesity, anti-infective, insulin inhibitor and anti-mycobacterial potential of NHDIPSA using *in silico* approaches. The rest of the lowest binding energy, inhibition constant and RMSD value of the docking complex were presented in Table 5. The stability of the protein–ligand complexes were not evaluated molecular dynamics simulations. Thus, the reported binding modes represent static interaction snapshots. Future studies will include MD simulations to assess dynamic stability.

**Table 4:** PASS online predicts (*P*_a_ > *P*_i_) of NHDIPSA

*P*_a_ (possibility activity)	*P*_i_ (possibility inactivity)	Activity
0.459	0.026	Antimycobacterial
0.242	0.162	Antibacterial
0.174	0.142	Anti-infective
0.310	0.085	Insulin inhibitor
0.207	0.120	Antiobesity
0.474	0.015	Antituberculosic
0.232	0.200	Antineoplastic
0.136	0.079	HIV-2 reverse transcriptcose inhibitor

**Table 5:** Protein and ligand interaction of NHDIPSA

Drug	Protein	Type of activity	Mode	Binding affinity (kcal mol^−1^)	Estimated inhibition constant *K*_i_ (µM)	RMSD
NHDIPSA	2ZCK	Antibacterial	1	−10.45	0.02196	67.89
			2	−8.59	0.5017	41.29
			3	−8.22	0.9435	76.24
			4	−8.12	1.12	70.5
			5	−7.94	1.5	66.16
			6	−7.81	1.88	65.89
			7	−7.73	2.15	72.42
			8	−7.56	2.85	71.44
			9	−7.41	3.72	61.25
			10	−7.26	4.74	70.57
NHDIPSA	6AIJ	Anti-obesity	1	−7.73	2.16	41.21
			2	−7.26	4.76	57.9
			3	−7.22	5.09	57.87
			4	−6.98	7.60	38.92
			5	−6.76	11.00	49.18
			6	−6.74	11.50	51.15
			7	−6.59	14.90	21.32
			8	−6.54	16.07	53.88
			9	−6.53	16.34	53.84
			10	−6.46	18.31	50.6
NHDIPSA	6IEY	Anti-infective	1	−7.47	3.34	78.73
			2	−7.18	5.42	74.45
			3	−7.07	6.59	55.08
			4	−6.72	11.82	61.07
			5	−6.69	12.51	51.01
			6	−6.26	25.73	60.82
			7	−6.25	26.40	58.01
			8	−6.2	28.37	48.87
			9	−6.18	29.53	56.82
			10	−5.49	94.01	59.76
NHDIPSA	6X4C	Insulin inhibitor	1	−9.14	0.201	513.57
			2	−8.61	0.488	531.04
			3	−8.33	0.786	513.82
			4	−8.19	0.948	530.14
			5	−8.07	1.23	530.31
			6	−7.94	1.51	525.74
			7	−7.89	1.66	519.12
			8	−7.65	2.48	527.4
			9	−7.41	3.72	520.49
			10	−7.34	4.15	516.42
NHDIPSA	7ENS	Anti mycobacterial	1	−9.43	0.122	48.81
			2	−9.15	0.194	34.91
			3	−9.13	2.09	34.84
			4	−7.75	2.14	35.75
			5	−7.74	3.19	64.38
			6	−7.5	3.21	36.13
			7	−7.49	3.75	63.34
			8	−7.28	4.36	63.2
			9	−7.4	4.89	47.76
			10	−7.31	5.31	46.86

To contextualize the docking results, the binding mode of NHDIPSA was compared with the reported binding regions of co-crystallized ligands for each protein target. Although direct re-docking of native ligands was not performed, NHDIPSA was found to occupy similar active site regions and to establish key intermolecular interactions, including hydrogen bonding and π–π stacking, with residues known to be critical for ligand binding. Therefore, the docking results are interpreted qualitatively in terms of structural complementarity rather than direct numerical comparison of binding energies.

#### 3.11.1 Docking of NHDIPSA with 2ZCK

The antibacterial agents are widely used to prevent or treat bacterial infection in humans and animals. The antibacterial protein 2ZCK was the subject of a simulation study in docking calculations. The grid size of the protein was 126 × 126 × 126 Å^3^, and its lowest inhibitory constant was 0.02196 µM. The lowest optimum binding energy for the target protein 2ZCK to the NHDIPSA was −10.45 kcal mol^−1^. In the 2D interaction diagram (Fig. 15), the green dotted lines represent conventional hydrogen bonds formed between the ligand's oxygen atom and the surrounding amino acid residues, at distances of 1.91 (THR-17), 2.26 (LYS-204), 3.06 (GLN-15), 2.61 (TYR-134), 2.82 (HIS-159), and 2.62 (LYS-201) angstroms.

#### 3.11.2 Docking of NHDIPSA with 6AIJ

The target protein's three-dimensional receptor structure (PDB ID: 6AIJ) was retrieved from the protein data bank. A 126 × 126 × 126 A^3^ grid was generated by means of the auto grid. The residue ILE-216 was reacted with hydrogen atom to form a hydrogen bond with the distance of 1.99 Å. The amino acid ala-223 FORM A π–sigma interaction between core of the phenyl ring and nitrogen atom with the distance of 3.42 and 4.86 Å. The amino acid ALA-212, ALA-198, ALA-215 and ILE-222 were forming a Pi–alkyl bond between the core of the phenyl ring with the distance of 3.71, 4.57, 4.91 and 5.02 Å respectively. From this analysis the global minimum energy was −7.73 kcal mol^−1^ and highest energy was −6.46 kcal mol^−1^ and the corresponding inhibition constant values ranges from minimum to maximum were 2.16–18.31 µM. The free energy, partition function, internal energy and entropy of this docking analysis was −1371.2 kcal mol^−1^, −6.88 kcal mol^−1^, 10.12 and 4.58 kcal mol^−1^ K^−1^.

#### 3.11.3 Docking of NHDIPSA with 6IEY

Simulations of molecular docking with the NHDIPSA complex in the 6IEY protein's active region showed a strong binding profile with many non-covalent stabilizing forces. To be more precise, the amino group's oxygen and hydrogen atoms made strong hydrogen bonds with the residues ALA-265, ARG-175 respectively, at bond distances of 2.22 Å and 2.45 Å. Interactions between alkyl and π–alkyl groups improved the complex's hydrophobic stability even more. The amino acid LUE-238 forms an unfavorable acceptor–acceptor interaction with oxygen atom in the distance of 2.465 Å. The amino acid PRO-240 make a π–alkyl interaction with core of phenyl ring in the distance of 4.29 and 5.13 Å. The residue ALA-173 constitutes a π–sigma bond with the core of the phenyl ring at the distance of 3.09, 3.21 and 5.09 Å. From this docking analysis the global minimum binding energy was −7.47 kcal mol^−1^ with inhibition constant of 3.34 µM and the highest binding energy was −5.49 Å kcal mol^−1^ with inhibition constant of 94.01 µM. This docking analysis carried out the following Lamarckian genetic algorithm (LGA) where the patrician function *Q* is 10.11, free energy A is approximately equal to −1370.79 kcal mol^−1^, the internal energy of this confirmation is *U* = −655 kcal mol^−1^ and the value of entropy is *s* = 4.58 kcal mol^−1^ K^−1^ with temperature of 298.15 K.

#### 3.11.4 Docking of NHDIPSA with 6X4C

The NHDIPSA complex was shown to have a stable, high-affinity binding profile inside the 6X4C protein's catalytic pocket according to molecular docking simulations. In particular, strong hydrogen bonds were formed between the oxygen and hydrogen atoms of the amino group of the ligand with the residues ILE-94, LYS-268 make a hydrogen bond with in the distance of 2.04 and 2.11 Å. The amino acid TRP-266 make a π–sulfur bond between SO_2_ and core of the phenyl ring with the distance of 3.64, 4.34 and 6.68 Å. The amino acid LYS-259, form a π–sigma bonding between core of the phenyl ring with the distance of 3.55 and 4.09 Å. From this analysis the global minimum energy was −9.14 kcal mol^−1^ and highest energy was −7.34 kcal mol^−1^ and the corresponding inhibition constant values ranges from minimum to maximum were 0.201–4.15 µM. The free energy, partition function, internal energy and entropy of this docking analysis was −1372.2 kcal mol^−1^, −8.06 kcal mol^−1^, 10.14 and 4.58 kcal mol^−1^ K^−1^.

#### 3.11.5 Docking of NHDIPSA with 7ENS

A strong binding profile defined by several non-covalent stabilizing forces was uncovered by molecular docking simulations of the NHDIPSA complex inside the active domain of the 7ENS protein (*Mycobacterium tuberculosis* InhA). The amino acid THR-17, GLN-15, LYS-204, TYR-134, HIS-159, LYS-201, THR-113 were formed hydrogen bond interaction with various atoms of the title compound with the distance of 1.90, 3.06, 2.26, 2.61, 2.52, 2.62, and 1.99 Å respectively. The amino acid ILE-54 make a π–alkyl bond with the distance of 5.18 and 5.42 Å with the core of the phenyl ring. The minimum binding energy of this docking pose was −9.43 kcal mol^−1^ and the maximum binding energy was −7.31 kcal mol^−1^ with the inhibition constant was 0.122 µM. The free energy, partition function, internal energy and entropy of this docking analysis was −1372.26 kcal mol^−1^, −8.02 kcal mol^−1^, 10.14 and 4.58 kcal mol^−1^ K^−1^.

## 4 Conclusion

In this work, the molecular framework and spectroscopic properties of the synthesized compound NHDIPSA were thoroughly studied by means of a combination of experimental and Density Functional Theory (DFT) calculations. The optimized molecular geometry can serve as a reliable structural basis for interpreting the experimental observation. This is also supported by vibrational analysis. The characteristic N–H and O–H stretching modes in the FT-IR spectrum (3306–3267 cm^−1^), are well reproduced in the Raman spectra and are in excellent agreement with theoretical predictions based on Potential Energy Distribution (PED) analysis. Similarly, the mid-frequency skeletal region displays good correspondence between the experimental and calculated bands associated with C=O stretching and sulfonyl (SO_2_) asymmetric and symmetric vibrations. The vibrational modes assigned to amide, sulfonyl, imidazole, aromatic and methylene functional groups were identified with good reliability with consistent IR and Raman intensities patterns.

Moreover, the experimental ^1^H and ^13^C NMR chemical shifts are in good agreement with theoretical values. Low RMSD values support the accuracy of the optimized molecular geometry. The observed shielding/deshielding trends of the amide linkage, aromatic rings and imidazole moiety are consistent with π-conjugative effects across the aromatic–imidazole–sulfonyl framework, providing a consistent interpretation over NMR, IR and Raman analyzes. Mass spectral studies provide further evidence for the proposed molecular framework with the molecular ion peak and characteristic fragmentation pathways typical for the conjugated aromatic and sulfonyl parts. In addition, the analysis of PES ensures the optimized conformation is a real minimum. Moreover, the topological analyzes of the electron density give more information about the bonding interactions and the pathways of delocalization of electrons.

Molecular docking studies were performed against selected biological target proteins (PDB IDs: 2ZCK, 6AIJ, 6IEY, 6X4C, and 7ENS) to qualitatively explore possible ligand–target interaction modes. The calculated binding energies indicate energetically favourable interactions, and the binding poses suggest that functional groups such as the amide, sulfonyl, imidazole, and conjugated aromatic moieties contribute to stabilizing contacts within the binding sites. Ramachandran plot analysis confirms the stereochemical integrity of the protein structures used for docking. These results are intended to provide preliminary insight into potential interaction patterns rather than to establish confirmed biological activity.

This study has several limitations. The docking protocol used a rigid-protein, flexible-ligand model that does not account for protein flexibility, solvent dynamics, or entropic contributions to binding, and no molecular dynamics simulation or MM/GBSA free-energy refinement was carried out to confirm complex stability. PASS predictions are derived from structural similarity to a training set of known bioactive compounds and report a probability estimate rather than a measured activity. Both approaches therefore generate structure-based hypotheses about possible target interactions, and neither substitutes for enzyme inhibition, antimicrobial, or cell-based experimental validation.

Overall, the strong agreement between experimental and theoretical results across infrared, Raman, nuclear magnetic resonance, mass spectrometry, PES, and topological analyses validate the structural and electronic characterization of NHDIPSA. The combined experimental and computational approach employed in this study offers a consistent and reliable framework for understanding the physicochemical properties of conjugated molecular systems. The biological relevance inferred from molecular docking and PASS analyses is exploratory in nature and requires further experimental validation. Nevertheless, the present findings provide a useful foundation for future theoretical and experimental investigations involving related molecular architectures and biological targets.

## Conflicts of interest

The authors declare no conflicts of interest.

## Supplementary Material

RA-OLF-D6RA05618K-s001

## Data Availability

The figures and tables data supporting this article have been included as part of the supplementary information (SI). Supplementary information is available. See DOI: https://doi.org/10.1039/d6ra05618k.
