# Supplementary material for: Spectroscopic characterization and DFT-based structural analysis of a novel sulfonyl-acetamide imidazole derivative with multi-target protein docking studies
Source: RSC Adv. 2026 Sep 10;16(43):53923–47. doi: 10.1039/d6ra05618k (PMC13559897; doi:10.1039/d6ra05618k)
Supplement: Supplementary file 1 [file RA-016-D6RA05618K-s001.pdf]

Supplementary Information (SI) for RSC Advances.
This journal is © The Royal Society of Chemistry 2026

Supplementary material

Spectroscopic Characterization and DFT-Based Structural Analysis of a Novel Sulfonyl-Acetamide Imidazole Derivative with Multi-Target Protein Docking Studies

R. Geetha[a,b], V. Balachandran[a,c*], R. Arulraj[d*], N. Thirughanasambantham[a]. Narayana[e,f], D. Mohan Radheep[g], A. Jayashree[e], V.S.K. Venkatachalapathy[h], Ponnadurai Ramasami[i,j]

[a] *Department of Physics, Arignar Anna Government Arts College (Affiliated to Bharathidasan University), Musiri, Tiruchirappalli 621211, India.*

[b] *Department of Physics, Sri Manakula Vinayagar Engineering College, Madagadipet, Puducherry 605107, India.*

[c] *Department of Physics, Subasakthi College of Arts and Science for Women, Sathiyamagalam, Kulithalai 639 120, Karur, Tamil Nadu, India.*

[d] *Department of Chemistry, Sri Manakula Vinayagar Engineering College, Madagadipet, Puducherry 605107, India.*

[e] *Department of Studies in Chemistry, Mangalore University, Mangalagangotri - 574 199, India.*

[f] *K.K. University, Bihar Sherif, Berauti, Bihar 803115, India.*

[g] *Department of Physics, Tamil Nadu Open University, Saidapet, Chennai 600015, India.*

[h] *Centre for Research and Development, Sri Manakula Vinayagar Engineering College, Madagadipet, Puducherry 605107, India.*

[i] *Computational Chemistry Group, Department of Chemistry, Faculty of Science, University of Mauritius, Réduit 80837, Mauritius*

[j] *Centre for Natural Product Research, Department of Chemical Sciences, University of Johannesburg, Doornfontein Campus, Johannesburg 2028, South Africa.*

**Corresponding Authors: Email ID: brsbala@rediffmail.com (V. Balachandran); rarulraj108@gmail.com (R. Arulraj); p.ramasami@uom.ac.mu (R. Ponnadurai)*

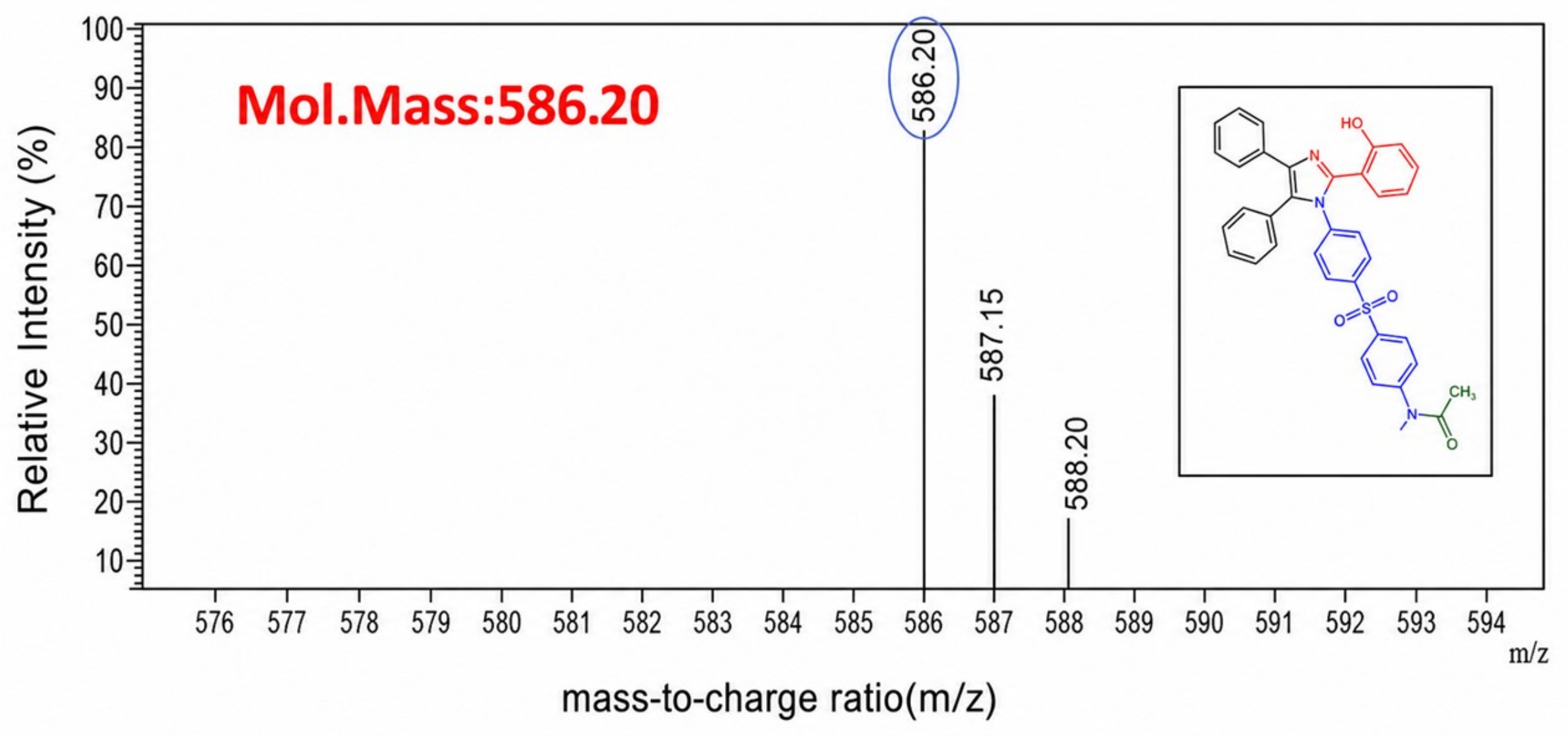

Fig. S1: Mass spectrum of NHDIPSA.

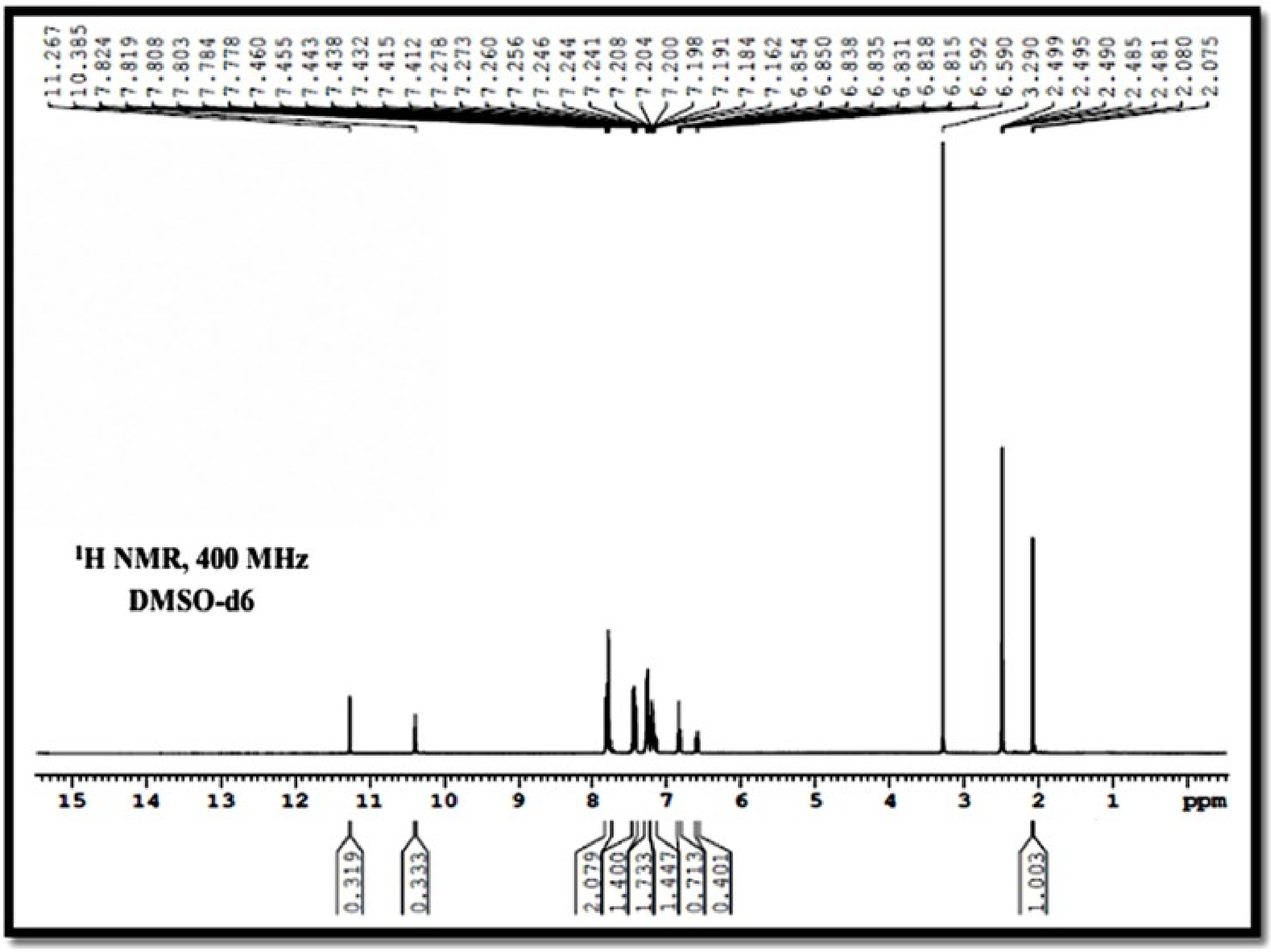

Fig. S2: ^{1}H-NMR spectrum of NHDIPSA.

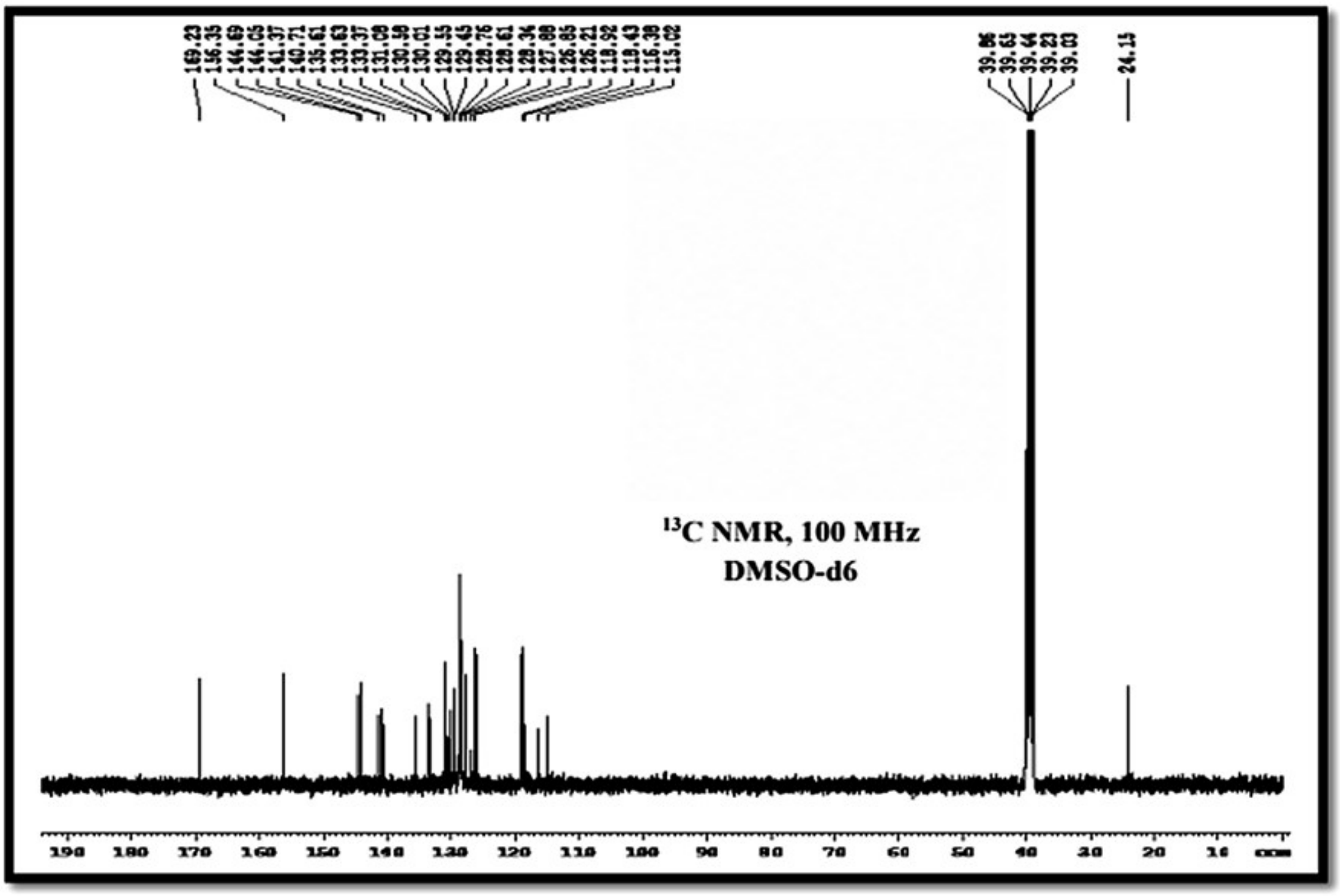

Fig. S3: ^{13}C-NMR spectrum of NHDIPSA.

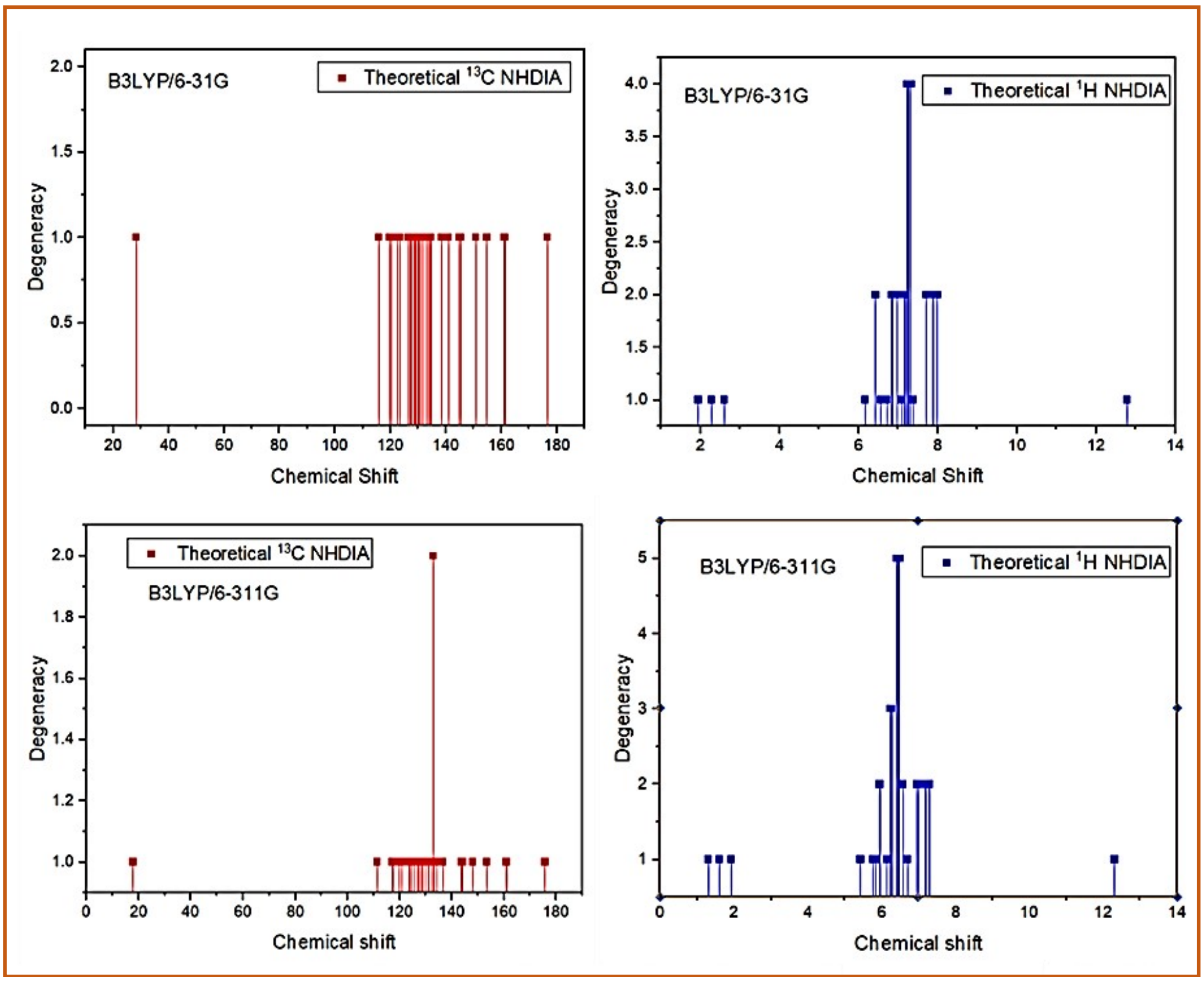

Fig. S4: 1D-Theoretical ^{13}C NMR and ^{1}H NMR chemical shifts for NHDIPSA.

Table S1: Optimized Geometric Parameters for NHDIPSA computed at B3LYP/6-31G(d,p)/6-311G(d,p)

Bond Length (Å)	B3LYP/6-31G(d,p)	B3LYP/6-311G(d,p)	Experimental*	Bond Angle (◦)	B3LYP/6-31G(d,p)	B3LYP/6-311G(d,p)	Experimental*	Dihedral Angle (◦)	B3LYP/6-31G(d,p)	B3LYP/6-311G(d,p)
**C1-N2**	1.42	1.42	1.44	**N2-C1-C5**	105.80	105.86	**110.10**	**C5-C1-N2-C3**	-1.36	-1.36
**C1-C5**	1.39	1.38	1.38	**N2-C1-C6**	122.89	122.70	**121.80**	**C5-C1-N2-C40**	176.56	176.99
**C1-C6**	1.48	1.48	1.44	**C5-C1-C6**	131.28	131.43	**127.60**	**C6-C1-N2-C3**	176.99	177.43
**N2-C3**	1.40	1.39	1.35	**C1-N2-C3**	107.74	107.70	**106.60**	**C6-C1-N2-C40**	-5.09	-4.22
**N2-C40**	1.44	1.44	1.48	**C1-N2-C40**	124.14	124.03	**123.50**	**N2-C1-C5-N4**	0.92	0.92
**C3-N4**	1.34	1.34	1.34	**C3-N2-C40**	128.08	128.25	**127.80**	**N2-C1-C5-C17**	-178.20	-178.59
**C3-C28**	1.46	1.46	1.42	**N2-C3-N4**	108.70	108.71	**110.80**	**C6-C1-C5-N4**	-177.24	-177.72
**N4-C5**	1.39	1.39	1.39	**N2-C3-C28**	129.37	129.24	**127.80**	**C6-C1-C5-C17**	3.64	2.77
**C5-C17**	1.47	1.47	1.48	**N4-C3-C28**	121.93	122.05	**120.80**	**N2-C1-C5-C17**	61.37	62.99
**C6-C7**	1.41	1.41	1.40	**C3-N4-C5**	108.85	108.82	**108.40**	**N2-C1-C5-C17**	-119.55	-118.07
**C6-C11**	1.41	1.41	1.39	**C1-C5-N4**	108.88	108.90	**108.40**	**C5-C1-C6-C7**	-120.74	-118.56
**C7-C8**	1.40	1.40	1.38	**C1-C5-C17**	130.80	130.89	**127.60**	**C5-C1-C6-C7**	58.34	60.38
**C7-H16**	1.08	1.08	1.01	**N4-C5-C17**	120.31	120.22	**120.00**	**C1-N2-C3-N4**	1.32	1.32
**C8-C9**	1.40	1.40	1.38	**C1-C6-C7**	121.61	121.49	**120.00**	**C1-N2-C3-C28**	-178.85	-178.85
**C8-H15**	1.09	1.08	1.01	**C1-C6-C11**	119.75	119.87	**120.00**	**C40-N2-C3-N4**	-176.49	-176.94
**C9-C10**	1.40	1.40	1.38	**C7-C6-C11**	118.64	118.63	**119.60**	**C40-N2-C3-C28**	3.33	2.89
**C9-H14**	1.09	1.08	1.01	**C6-C7-C8**	120.60	120.62	**120.00**	**C1-N2-C40-C41**	70.13	70.87
**C10-C11**	1.40	1.40	1.39	**C6-C7-H16**	119.59	119.57	**120.00**	**C1-N2-C40-C45**	-109.32	-108.80
**C10-H13**	1.09	1.08	1.01	**C8-C7-H16**	119.80	119.80	**120.00**	**C3-N2-C40-C41**	-112.39	-111.13
**C11-H12**	1.08	1.08	1.01	**C7-C8-C9**	120.23	120.21	**120.00**	**C3-N2-C40-C45**	68.17	69.20
**C17-C18**	1.41	1.41	1.40	**C7-C8-H15**	119.67	119.69	**120.00**	**N2-C3-N4-C5**	-0.75	-0.75
**C17-C22**	1.41	1.41	1.40	**C9-C8-H15**	120.09	120.10	**120.00**	**C28-C3-N4-C5**	179.41	179.41
**C18-C19**	1.40	1.40	1.40	**C8-C9-C10**	119.70	119.69	**119.20**	**N2-C3-C28-C29**	-166.73	-166.39
**C18-H27**	1.08	1.08	1.01	**C8-C9-H14**	120.16	120.16	**120.00**	**N2-C3-C28-C33**	14.61	15.11
**C19-C20**	1.40	1.40	1.40	**C10-C9-H14**	120.14	120.15	**120.00**	**N4-C3-C28-C29**	13.07	13.42
**C19-H26**	1.09	1.08	1.01	**C9-C10-C11**	120.19	120.18	**120.00**	**N4-C3-C28-C33**	-165.59	-165.07

**C20-C21**	1.40	1.40	1.39	**C9-C10-H13**	120.12	120.12	**120.01**	**C3-N4-C5-C1**	-0.13	-0.13
**C20-H25**	1.09	1.08	1.01	**C11-C10-H13**	119.69	119.70	**120.00**	**C3-N4-C5-C17**	179.11	179.45
**C21-C22**	1.40	1.39	1.40	**C6-C11-C10**	120.64	120.66	**120.00**	**C1-C5-C17-C18**	26.73	26.99
**C21-H24**	1.09	1.08	1.01	**C6-C11-H12**	119.18	119.22	**120.00**	**C1-C5-C17-C22**	-154.67	-154.26
**C22-H23**	1.08	1.08	1.01	**C10-C11-H12**	120.18	120.12	**120.00**	**N4-C5-C17-C18**	-152.31	-152.47
**C28-C29**	1.43	1.43	1.40	**C5-C17-C18**	122.14	122.19	**121.80**	**N4-C5-C17-C22**	26.29	26.27
**C28-C33**	1.41	1.41	1.40	**C5-C17-C22**	119.28	119.27	**120.10**	**C1-C6-C7-C8**	179.22	179.00
**C29-C30**	1.40	1.40	1.39	**C18-C17-C22**	118.57	118.53	**120.00**	**C1-C6-C7-H16**	0.15	0.13
**C29-O38**	1.37	1.37	1.35	**C17-C18-C19**	120.57	120.59	**120.20**	**C11-C6-C7-C8**	0.13	0.05
**C30-C31**	1.39	1.39	1.40	**C17-C18-H27**	119.88	119.90	**120.10**	**C11-C6-C7-H16**	-178.94	-178.82
**C30-H37**	1.08	1.08	1.01	**C19-C18-H27**	119.55	119.50	**120.00**	**C1-C6-C11-C10**	-178.92	-178.73
**C31-C32**	1.40	1.40	1.38	**C18-C19-C20**	120.39	120.38	**120.70**	**C1-C6-C11-H12**	0.65	0.73
**C31-H36**	1.09	1.08	1.01	**C18-C19-H26**	119.55	119.56	**120.10**	**C7-C6-C11-C10**	0.19	0.23
**C32-C33**	1.39	1.39	140	**C20-C19-H26**	120.06	120.05	**120.00**	**C7-C6-C11-H12**	179.76	179.70
**C32-H35**	1.08	1.08	1.01	**C19-C20-C21**	119.50	119.50	**122.30**	**C6-C7-C8-C9**	-0.27	-0.23
**C33-H34**	1.08	1.08	1.01	**C19-20-H25**	120.24	120.23	**120.00**	**C6-C7-C8-H15**	-179.64	-179.57
**O38-H39**	1.01	1.00	0.96	**C21-C20-H25**	120.26	120.27	**120.00**	**H16-C7-C8-C9**	178.79	178.64
**C40-C41**	1.40	1.40	1.40	**C20-C21-C22**	120.29	120.30	**120.00**	**H16-C7-C8-H15**	-0.57	-0.69
**C40-C45**	1.40	1.40	1.40	**C20-C21-H24**	120.10	120.08	**120.00**	**C7-C8-C9-C10**	0.09	0.13
**C41-C42**	1.40	1.40	1.40	**C22-C21-H24**	119.62	119.62	**120.00**	**C7-C8-C9-H14**	-179.64	-179.58
**C41-H49**	1.08	1.08	1.05	**C17-C22-C21**	120.68	120.70	**120.40**	**H15-C8-C9-C10**	179.45	179.46
**C42-C43**	1.39	1.39	1.40	**C17-C22-H23**	119.02	118.97	**119.20**	**H15-C8-C9-H14**	-0.28	-0.25
**C42-H48**	1.08	1.08	1.05	**C21-C22-H23**	120.30	120.33	**120.00**	**C8-C9-C10-C11**	0.22	0.16
**C43-C44**	1.39	1.39	1.40	**C3-C28-C29**	117.75	118.02	**120.00**	**C8-C9-C10-H13**	-179.92	-179.87
**C43-S50**	1.88	1.89	1.91	**C3-C28-C33**	124.33	124.18	**120.60**	**H14-C9-C10-C11**	179.95	179.87
**C44-C45**	1.40	1.40	1.39	**C29-C28-C33**	117.90	117.78	**119.60**	**H14-C9-C10-H13**	-0.19	-0.16
**C44-H47**	1.08	1.08	1.05	**C28-C29-C30**	119.99	120.09	**120.00**	**C9-C10-C11-C6**	-0.37	-0.34
**C45-H46**	1.08	1.08	1.01	**C28-C29-O38**	122.74	122.85	**120.30**	**C9-C10-C11-H12**	-179.93	-179.80
**S50-O51**	1.65	1.65	1.58	**C30-C29O38**	117.27	117.06	**119.90**	**H13-C10-C11-C6**	179.77	179.69
**S50-O52**	1.65	1.65	1.58	**C29-C30-C31**	120.63	120.64	**120.00**	**H13-C10-C11-H12**	0.21	0.23
**S50-C53**	1.87	1.88	1.78	**C29-C30-H37**	117.48	117.45	**119.60**	**C5-C17-C18-C19**	179.36	179.41

**C53-C54**	1.39	1.39	1.35	**C31-C30-H37**	121.89	121.90	**121.60**	**C5-C17-C18-H27**	0.42	0.43
**C53-C58**	1.39	1.39	1.35	**C30-C31-C32**	120.14	120.11	**120.00**	**C22-C17-C18-C19**	0.75	0.65
**C54-C55**	1.39	1.39	1.35	**C30-C31-H36**	119.75	119.75	**120.00**	**C22-C17-C18-H27**	-178.19	-178.33
**C54-H59**	1.08	1.08	1.05	**C32-C31-H36**	120.10	120.13	**120.00**	**C5-C17-C22-C21**	-179.25	-179.34
**C55-C56**	1.41	1.41	1.40	**C31-C32-C33**	119.75	119.74	**120.00**	**C5-C17-C22-H23**	0.91	0.73
**C55-H60**	1.09	1.08	1.05	**C31-C32-H35**	120.47	120.47	**120.00**	**C18-C17-C22-C21**	-0.61	-0.55
**C56-C57**	1.41	1.41	1.40	**C33-C32-H35**	119.78	119.78	**119.50**	**C18-C17-C22-H23**	179.55	179.52
**C56-N63**	1.41	1.41	1.39	**C28-C33-C32**	121.51	121.56	**121.80**	**C17-C18-C19-C20**	-0.36	-0.28
**C57-C58**	1.40	1.40	1.39	**C28-C33-H34**	119.92	119.90	**120.00**	**C17-C18-C19-H26**	-179.70	-179.68
**C57-H62**	1.08	1.08	1.05	**C32-C33-H34**	118.57	118.53	**118.80**	**H27-C18-C19-C20**	178.58	178.70
**C58-H61**	1.08	1.08	1.05	**C29-O38-H39**	109.19	109.58	**106.60**	**H27-C18-C19-H26**	-0.75	-0.69
**N63-H64**	1.01	1.01	0.95	**N2-C40-C41**	119.67	119.65	**120.00**	**C18-C19-C20-C21**	-0.19	-0.20
**N63-C65**	1.39	1.39	1.40	**N2-C40-C45**	119.51	119.54	**120.00**	**C18-C19-C20-H25**	-179.84	-179.85
**C65-O66**	1.25	1.25	1.20	**C41-C40-C45**	120.82	120.81	**12010**	**H26-C19-C20-C21**	179.14	179.19
**C65-C67**	1.51	1.51	1.45	**C40-C41-C42**	119.77	119.82	**120.00**	**H26-C19-C20-H25**	-0.50	-0.45
**C67-H68**	1.09	1.09	1.05	**C40-C41-H49**	119.66	119.69	**120.10**	**C19-C20-C21-C22**	0.34	0.31
**C67-H69**	1.10	1.09	1.05	**C42-C41-H49**	120.56	120.49	**120.60**	**C19-C20-C21-H24**	-179.71	-179.71
**C67-H70**	1.09	1.09	1.05	**C41-C42-C43**	118.12	117.94	**118.80**	**H25-C20-C21-C22**	179.98	179.95
				**C41-C42-H48**	121.52	121.63	**121.60**	**H25-C20-C21-H24**	-0.06	-0.06
				**C43-C42-H48**	120.32	120.39	**120.10**	**C20-C21-C22-C17**	0.07	0.08
				**C42-C43-C44**	123.39	123.67	**122.30**	**C20-C21-C22-H23**	179.90	-180.00
				**C42-C43-S50**	118.34	118.15	**119.40**	**H24-C21-C22-C17**	-179.89	-179.91
				**C44-C43-S50**	118.23	118.14	**120.40**	**H24-C21-C22-H23**	-0.05	0.02
				**C43-C44-C45**	118.11	117.93	**118.80**	**C3-C28-C29-C30**	177.98	178.19
				**C43-C44-H47**	120.27	120.39	**120.10**	**C3-C28-C29-O38**	-2.22	-1.82
				**C45-C44-H47**	121.60	121.66	**121.60**	**C33-C28-C29-C30**	-3.27	-3.22
				**C40-C45-C44**	119.77	119.82	**119.40**	**C33-C28-C29-O38**	176.53	176.78
				**C40-C45-H46**	119.65	119.69	**119.60**	**C3-C28-C33-C32**	-178.88	-179.08
				**C44-C45-H46**	120.57	120.48	**120.30**	**C3-C28-C33-H34**	2.31	1.89
				**C43-S50-O51**	106.16	105.86	**106.60**	**C29-C28-C33-C32**	2.47	2.42
				**C43-S50-O52**	106.35	105.96	**160.80**	**C29-C28-C33-H34**	-176.34	-176.61

**O51-S50-C53**	106.79	106.35	**106.10**	**C28-C29-C30-C31**	1.86	1.88
**O52-S50-C53**	106.68	106.32	**106.00**	**C28-C29-C30-H37**	-179.06	-179.10
**S50-C53-C54**	118.70	118.49	**119.60**	**O38-C29-C30-C31**	-177.95	-178.12
**S50-C53-C58**	118.58	118.46	**119.10**	**O38-C29-C30-H37**	1.13	0.90
**C54-C53-C58**	122.65	122.99	**122.30**	**C28-C29-O38-H39**	-5.15	-5.39
**C53-C54-C55**	118.26	118.12	**118.00**	**C30-C29-O38-H39**	174.65	174.61
**C53-C54-H59**	120.42	120.44	**120.10**	**C29-C30-C31-C32**	0.48	0.38
**C55-C54-H59**	121.31	121.42	**121.80**	**C29-C30-C31-H36**	179.55	179.54
**C54-C55-C56**	120.79	120.71	**120.40**	**H37-C30-C31-C32**	-178.56	-178.59
**C54-C55-H60**	119.76	119.77	**120.00**	**H37-C30-C31-H36**	0.51	0.57
**C56-C55-H60**	119.45	119.52	**119.80**	**C30-C31-C32-C33**	-1.32	-1.22
**C55-C56-C57**	119.23	119.30	**119.80**	**C30-C31-C32-H35**	178.33	178.42
**C55-C56-N63**	117.35	117.89	**118.40**	**H36-C31-C32-C33**	179.61	179.62
**C57-C56-N63**	123.36	122.74	**122.30**	**H37-C30-C31-H36**	-0.73	-0.75
**C56-C57-C58**	120.20	120.27	**120.10**	**C31-C32-C33-C28**	-0.20	-0.23
**C56-C57-H62**	120.67	120.47	**120.10**	**C31-C32-C33-H34**	178.62	178.81
**C58-C57-H62**	119.08	119.21	**120.00**	**H35-C32-C33-C28**	-179.86	-179.87
**C53-C58-C57**	118.80	118.53	**118.80**	**H35-C32-C33-H34**	-1.03	-0.83
**C53-C58-H61**	120.07	120.21	**120.00**	**N2-C40-C41-C42**	-179.32	-179.55
**C57-C58-H61**	121.06	121.18	**121.00**	**N2-C40-C41-H49**	1.36	1.23
**C56-N63-H64**	115.93	116.31	**116.80**	**C45-C40-C41-C42**	0.12	0.11
**C56-N63-C65**	132.75	132.02	**127.60**	**C45-C40-C41-H49**	-179.20	-179.11
**H64-N63-C65**	111.24	111.58	**110.10**	**N2-C40-C45-C44**	179.71	179.96
**N63-C65-O66**	118.36	118.36	**119.40**	**N2-C40-C45-H46**	-1.64	-1.38
**N63-C65-C67**	119.93	119.91	**119.60**	**C41-C40-C45-C44**	0.27	0.30
**O66-C65-C67**	121.69	121.71	**121.60**	**C41-C40-C45-H46**	178.92	178.95
**C65-C67-H68**	112.86	113.10	**113.20**	**C40-C41-C42-C43**	0.24	0.17
**C65-C67-H69**	110.85	110.82	**110.10**	**C40-C41-C42-H48**	-177.57	-177.58
**C65-C67-H70**	107.30	107.32	**106.60**	**H49-C41-C42-C43**	179.56	179.39
**H68-C67-H69**	108.33	108.28	**108.40**	**H49-C41-C42-H48**	1.75	1.64
**H68-C67-H70**	109.77	109.70	**110.10**	**C41-C42-C43-C44**	-1.04	-0.91

**H69-C67-H70**	107.61	107.47	**108.40**	**C41-C42-C43-S50**	-178.78	-178.52
				**H48-C42-C43-C44**	176.80	176.87
				**H48-C42-C43-S50**	-0.94	-0.74
				**C42-C43-C44-C45**	1.43	1.31
				**C42-C43-C44-H47**	-176.82	-176.84
				**S50-C43-C44-C45**	179.17	178.92
				**S50-C43-C44-H47**	0.92	0.77
				**C42-C43-S50-O51**	155.92	156.29
				**C42-C43-S50-O52**	23.20	21.62
				**C44-C43-S50-O51**	-21.94	-21.46
				**C44-C43-S50-O52**	-154.66	-156.13
				**C42-C43-C53-C54**	-143.94	-145.01
				**C42-C43-C53-C58**	1.97	0.79
				**C44-C43-C53-C54**	2.12	0.98
				**C44-C43-C53-C58**	148.03	146.79
				**C43-C44-C45-C40**	-1.00	-0.97
				**C43-C44-C45-H46**	-179.64	-179.61
				**H47-C44-C45-C40**	177.22	177.16
				**H47-C44-C45-H46**	-1.42	-1.49
				**O51-S50-C53-C54**	23.85	22.00
				**O51-S50-C53-C58**	-153.28	-155.11
				**O52-S50-C53-C54**	157.22	157.27
				**O52-S50-C53-C58**	-19.91	-19.84
				**S50-C53-C54-C55**	-179.49	-179.42
				**S50-C53-C54-H59**	-0.81	-0.71
				**C58-C53-C54-C55**	-2.48	-2.44
				**C58-C53-C54-H59**	176.20	176.26
				**S50-C53-C58-C57**	178.63	178.48
				**S50-C53-C58-H61**	1.65	1.61
				**C54-C53-C58-C57**	1.62	1.50
				**C54-C53-C58-H61**	-175.36	-175.36

**C53-C54-C55-C56**	0.89	0.93
**C53-C54-C55-H60**	-179.05	-178.89
**H59-C54-C55-C56**	-177.77	-177.77
**H59-C54-C55-H60**	2.28	2.42
**C54-C55-C56-C57**	1.46	1.43
**C54-C55-C56-N63**	178.64	178.45
**H60-C55-C56-C57**	-178.59	-178.76
**H60-C55-C56-N63**	-1.41	-1.74
**C55-C56-C57-C58**	-2.35	-2.39
**C55-C56-C57-H62**	175.07	175.03
**N63-C56-C57-C58**	-179.35	-179.26
**N63-C56-C57-H62**	-1.93	-1.84
**C55-C56-N63-H64**	-24.22	-28.97
**C55-C56-N63-C65**	152.21	147.27
**C57-C56-N63-H64**	152.84	147.94
**C57-C56-N63-C65**	-30.73	-35.82
**C56-C57-C58-C53**	0.85	0.97
**C56-C57-C58-H61**	177.80	177.80
**H62-C57-C58-C53**	-176.61	-176.48
**H62-C57-C58-H61**	0.34	0.35
**C56-N63-C65-O66**	178.55	178.40
**C56-N63-C65-C67**	-3.08	-3.34
**H64-N63-C65-O66**	-4.90	-5.22
**H64-N63-C65-C67**	173.48	173.04
**N63-C65-C67-H68**	-35.91	-33.29
**N63-C65-C67-H69**	85.82	88.51
**N63-C65-C67-H70**	-156.95	-154.42
**O66-C65-C67-H68**	142.41	144.92
**O66-C65-C67-H69**	-95.86	-93.28
**O66-C65-C67-H70**	21.37	23.79

Table S2: Experimental and calculated ^{13}C-NMR and ^{1}H-NMR chemical shifts for NHDIPSA.

Atoms	B3LYP/6–31G DMSO	B3LYP/6–311G DMSO	Δδ (6-31G)	Experimental (ppm)	Δδ (6-311G)
	Carbon-13 (RMSD)		**3.16 ppm**		**3.10 ppm**
**65C**	176.55	175.67	**7.32**	169.23	**6.44**
**43C**	161.38	161.22	**5.03**	156.35	**4.87**
**29C**	161.14	160.86	**4.79**	156.35	**4.51**
**53C**	154.80	153.34	**10.11**	144.69	**8.65**
**3C**	150.82	147.95	**6.13**	144.69	**3.26**
**40C**	145.44	144.12	**0.75**	144.69	**0.57**
**56C**	144.98	143.74	**3.61**	141.37	**2.37**
**5C**	140.95	136.65	**0.24**	140.71	**4.06**
**1C**	138.65	134.18	**3.04**	135.61	**1.43**
**45C**	134.52	133.23	**1.09**	135.61	**2.38**
**17C**	134.30	132.88	**0.67**	133.63	**0.75**
**11C**	133.82	132.83	**0.19**	133.63	**0.80**
**41C**	133.69	132.26	**0.06**	133.63	**1.37**
**7C**	132.96	131.33	**0.41**	133.37	**2.04**
**6C**	131.83	130.84	**0.75**	131.08	**0.24**
**31C**	131.26	129.97	**0.18**	131.08	**1.11**
**10C**	130.46	129.01	**0.12**	130.58	**1.57**
**9C**	130.25	128.86	**0.24**	130.01	**1.15**
**21C**	130.09	128.41	**0.08**	130.01	**1.60**
**8C**	129.75	127.96	**0.20**	129.55	**1.59**
**22C**	129.18	127.34	**0.27**	129.45	**2.11**
**19C**	128.98	127.25	**0.22**	128.76	**1.51**
**18C**	128.73	127.09	**0.12**	128.61	**1.52**
**20C**	128.55	126.97	**0.19**	128.36	**1.39**
**33C**	127.79	125.49	**0.09**	127.88	**2.39**
**44C**	127.18	124.59	**0.70**	127.88	**3.29**
**42C**	127.04	124.50	**0.84**	127.88	**3.38**
**58C**	126.45	123.80	**0.40**	126.85	**3.05**
**54C**	126.28	123.61	**0.07**	126.21	**2.60**
**55C**	123.49	120.88	**4.57**	118.92	**1.96**

**57C**	122.42	119.84	**3.99**	118.43	**1.41**
**30C**	120.21	117.58	**3.78**	116.43	**1.15**
**32C**	119.71	117.16	**3.33**	116.38	**0.78**
**28C**	115.76	111.30	**0.74**	115.02	**3.72**
**67C**	28.37	17.78	**4.22**	24.15	**6.37**
	**Proton-1 (RMSD)**		**0.45 ppm**		**1.03 ppm**
**39H**	12.78	12.00	**1.52**	11.26	**0.74**
**23H**	7.99	7.00	**0.17**	7.82	**0.82**
**47H**	7.99	7.00	**0.18**	7.81	**0.81**
**61H**	7.89	7.00	**0.09**	7.8	**0.80**
**48H**	7.87	7.00	**0.07**	7.8	**0.80**
**46H**	7.71	7.00	**0.07**	7.78	**0.78**
**59H**	7.70	7.00	**0.07**	7.77	**0.77**
**24H**	7.38	7.00	**0.08**	7.46	**0.46**
**12H**	7.32	7.00	**0.13**	7.45	**0.45**
**13H**	7.28	7.00	**0.16**	7.44	**0.44**
**49H**	7.25	6.00	**0.18**	7.43	**1.43**
**62H**	7.23	6.00	**0.20**	7.43	**1.43**
**14H**	7.17	6.00	**0.24**	7.41	**1.41**
**25H**	7.14	6.00	**0.27**	7.41	**1.41**
**36H**	7.08	6.00	**0.19**	7.27	**1.27**
**60H**	6.96	6.00	**0.31**	7.27	**1.27**
**26H**	6.96	6.00	**0.30**	7.26	**1.26**
**37H**	6.85	6.00	**0.40**	7.25	**1.25**
**15H**	6.84	6.00	**0.40**	7.24	**1.24**
**27H**	6.72	6.00	**0.52**	7.24	**1.24**
**16H**	6.55	6.00	**0.69**	7.24	**1.24**
**35H**	6.43	6.00	**0.77**	7.2	**1.20**
**64H**	6.41	6.00	**0.78**	7.19	**1.19**
**34H**	6.16	6.00	**0.43**	6.59	**0.59**
**69H**	2.59	2.00	**0.10**	2.49	**0.49**
**68H**	2.28	2.00	**0.20**	2.48	**0.48**
**70H**	1.94	1.00	**0.13**	2.07	**1.07**

Table S3: Vibrational assignments of NHDIPSA

S. No	Observed wavenumbers (cm^{-1})		Calculated wavenumbers (cm^{-1})		Vibrational assignments (%PED)
	FT-IR	FT-Raman	B3LYP/ 6-31G(d,p)	B3LYP/ 6-311G(d,p)	
1.	3306		3312	3310	νOH (98)
2.	3267		3275	3271	νNH(98)
3.	3193		3198	3195	νCH (97)
4.	3114		3119	3117	νCH (98)
5.		3065	3073	3070	νCH (97)
6.	3059		3066	3062	νCH (97)
7.			3047	3045	νCH (98)
8.			3038	3035	νCH (99)
9.			3021	3018	νCH (98)
10.		3006	3013	3011	νCH (97)
11.			3005	3001	νCH (99)
12.			2998	2996	νCH (98)
13.			2985	2980	νCH (98)
14.			2974	2968	νCH (97)
15.			2952	2947	νCH (98)
16.			2934	2930	νCH (99)
17.			2928	2925	νCH (97)
18.			2921	2918	νCH (99)
19.		2931	2915	2909	νCH (98)
20.	2920		2908	2901	νCH (97)
21.			2900	2895	νCH (97)
22.			2894	2888	νCH (98)
23.			2889	2881	$\nu_{ass}CH_3$ (97)
24.			2884	2880	νCH (98)
25.			2877	2872	νCH (97)
26.			2870	2865	$\nu_{ass}CH_3$ (97)
27.	2851		2860	2855	$\nu_{ss}CH_3$ (97)
28.	1675	1674	1682	1678	νCO(65), δCC(21), δCN(10)
29.			1671	1669	νCC(65), δCH(20), δCO(12)

30.			1658	1654	νCC(64), δCH(15), δCO(10)
31.			1642	1637	νCC(69), δCH(20), δCO(10)
32.			1627	1625	νCC(75), δCH(19)
33.		1606	1615	1610	νCC(73), δCH(17)
34.			1604	1601	δCH(74), νCC(15)
35.	1589		1593	1590	νCC(73), δCH(18)
36.	1582		1588	1583	νCC(72), δCH(17)
37.			1575	1571	νCC(72), δCH(15)
38.		1561	1569	1563	νCC (76), δCH (12), δCO(10)
39.			1553	1551	δCH(74)
40.			1545	1540	δCH(74)
41.			1539	1532	δCH(75)
42.			1527	1524	δCH (74)
43.		1500	1511	1507	νCN(71)
44.			1503	1501	δCH(75), νCC(16)
45.	1489		1492	1488	δCH(71), νCC(15)
46.		1469	1476	1475	νCC (73), δCH(18)
47.			1463	1458	$\delta_{opb}CH_3$(84)
48.	1445		1452	1450	δCH(75), νCC(12)
49.			1439	1435	δCH (76), νCC(10)
50.			1423	1418	$\delta_{ipb}CH_3$(80)
51.	1401	1402	1412	1408	δCH(72), νCC(12)
52.			1401	1398	$\delta_{sb}CH_3$(75), δCC(12)
53.			1394	1392	νCC(72), $\delta_{sb}CH_3$(12)
54.	1379	1375	1382	1381	νCC(70), δCH(18), δCN(10)
55.			1375	1370	νCN(76), δCH(20)
56.			1364	1361	δCH(72)
57.			1359	1356	δCH(75), δCN(12)
58.			1345	1340	δCH(68), νCC(180), δCC(10)
59.	1328		1339	1337	νCC(65), νCH(18), δCC(10)
60.		1322	1329	1325	νCC(64), νCH(15),

					δCC(12)
61.			1322	1320	δCH(68), νCC(12)
62.		1306	1317	1313	δCH(65), νCC(12)
63.	1295		1307	1304	δCH(68)
64.			1293	1291	νCC(65), δCH(12)
65.			1284	1280	νCO(68), δCH(13)
66.	1258	1260	1267	1263	νCN(68), δCC(17)
67.			1264	1260	νCN(70), νCC(21)
68.			1257	1253	δCH(65)
69.		1241	1250	1248	δCH(66)
70.			1242	1240	δCH(70)
71.			1231	1228	δCH(72)
72.			1224	1218	δCH(76)
73.			1215	1211	δCH(72)
74.			1206	1204	δCH(76)
75.			1198	1195	δCH(75)
76.			1187	1182	δOH(75), δCH(10)
77.		1172	1179	1175	νCN(63), νCC(20), δCH(12)
78.	1161		1167	1165	$\delta_{opr}CH_3$(66), νCN(16), δCO(10)
79.		1155	1161	1158	δCH(65), νCN(15), δCO(12)
80.			1155	1151	δCH(72)
81.			1149	1145	νCN(69), δCH(15)
82.			1142	1140	νCN(68), δCH(18)
83.			1130	1126	νCH(65), δOH(18), νCN(12)
84.	1104	1103	1111	1108	δCH(68)
85.			1105	1100	δCH(65)
86.			1092	1090	νCS(60), νCC(20), δCH(12)
87.	1073		1083	1081	νCS(60), νCC(20), δCH(12)
88.		1061	1075	1070	δ_{ring}(68), νCS(12)
89.			1069	1062	δ_{ring}(68), δCH(15)
90.			1058	1052	δ_{ring}(68), δCH(15)
91.			1042	1040	$\delta_{ipr}CH_3$(68)

92.			1037	1034	δ_{ring}(65), δCH(12)
93.	1017	1020	1028	1025	δ_{ring}(66), δCH(12)
94.			1019	1016	δ_{ring}(65), νCS(16), δCH(10)
95.			1015	1010	δ_{ring}(66), νCC(18), δCH(10)
96.		999	1008	1002	γCH(65)
97.		982	988	985	γCH(65)
98.	972		975	971	γCH(65)
99.			964	962	γCCH_3(68), γCH(12)
100.			955	950	γCH(65)
101.			947	942	γCH(65)
102.			938	935	γCH(65)
103.			925	921	γCH(65)
104.			917	915	γCH(65)
105.			908	906	γCH(65)
106.			892	888	γCH(65)
107.			877	875	γCH(65)
108.	841		851	848	δ_{ring}(62)
109.			844	840	γCH(63)
110.			840	835	$\nu_{ass}SO_2$(70)
111.		829	832	827	γCH(63)
112.			817	814	γCH(65)
113.			809	802	γCH(65)
114.			796	790	γCH(65)
115.	780	783	789	784	γCH(65)
116.			780	777	γCH(63)
117.		767	772	770	γCH(63)
118.			768	765	γCH(65)
119.	755		759	757	γCH(65)
120.			751	750	γCH(65)
121.			748	745	δ_{ring}(68)
122.		735	740	738	γCH(65)
123.			734	731	γCH(65)
124.			725	720	$\nu_{ss}SO_2$(68)
125.			716	712	γCH(65)

126.	704		710	704	δ_{ring}(63)
127.			696	692	δ_{ring} (63)
128.			689	682	γCC(58)
129.			675	670	γCN(60)
130.			668	662	δ_{ring} (66)
131.	654		660	653	δ_{ring}(65)
132.			651	648	γCH(63)
133.			642	640	δ_{ring}(65)
134.	623	624	630	627	γ_{ring}(62)
135.			619	612	γ_{ring}(62)
136.			605	599	δCN(66), δ_{ring}(21)
137.	590		596	592	δ_{ring}(63)
138.			590	585	δ_{ring}(63)
139.			582	580	γCO(59)
140.			575	569	δ_{ring}(65)
141.	558		562	558	δ_{ring}(65)
142.			547	542	δ_{ring}(65)
143.			532	530	δ_{ring}(65)
144.			521	517	δ_{ring}(65)
145.			506	500	δCN(67), δCC(16)
146.			497	493	δCO(65), δCC(20)
147.			488	485	γ_{ring}(59)
148.			481	480	γ_{ring}(58)
149.			479	475	γ_{ring}(58)
150.			467	462	γ_{ring}(58)
151.			452	450	γ_{ring}(57)
152.			441	438	δCC(63)
153.			429	425	δCS(62)
154.			415	407	δ_{ring}(62)
155.			401	399	δNN(63)
156.			385	381	γCC(58)
157.		372	376	370	γCO(58), γ_{ring}(13)
158.			358	354	γ_{ring}(57)
159.			346	340	γ_{ring}(57)
160.			337	333	γ_{ring}(57)

161.		328	326	γ_{ring}(57)
162.		318	315	γ_{ring}(57)
163.		305	304	δCO(65), δ_{ring}(18)
164.		299	296	δCS (59), δ_{ring}(21)
165.		292	289	$\delta_{sciss}SO_2$(63)
166.		279	274	γCS(55), γOH(19)
167.		267	265	γOH(72)
168.	251	254	250	γCC(62)
169.		245	240	γCC(60)
170.		237	231	γCN(58)
171.		231	226	γCN(58)
172.		218	214	δ SO(68)
173.		209	206	$\delta_{rock}SO_2$(59)
174.		198	194	δ SO(68)
175.		185	180	δ_{ring}(66)
176.		177	174	δCC(62)
177.		163	159	γ_{ring}(59)
178.	153	157	151	δ_{ring}(65)
179.		143	138	δ_{ring}(65)
180.		136	130	δ_{ring}(52)
181.		128	121	τSO_2(54)
182.	112	115	111	γSO(50), γ_{ring}(18)
183.		109	105	δ_{ring}(52)
184.		103	101	δ_{ring}(52)
185.	94	98	96	γCC(48)
186.		95	91	γCC(48)
187.		90	88	γCC(48)
188.		88	85	γ_{Wagg} SO_2 (47)
189.		82	80	τCH_3(48)
190.		79	75	δCSC(47)
191.	72	75	71	γCC(48)
192.		70	68	γCC(47)
193.		66	65	γCC(47)
194.		61	57	γ_{ring} (45)
195.		55	52	γCC(48)

196.	52	49	γCC(48)
197.	49	44	γ_{ring} (48)
198.	42	40	δCC(45)
199.	36	35	τCC(46)
200.	31	29	τCC(45)
201.	25	21	τCC(46)
202.	18	14	γCC(46)
203.	12	10	τ CC(45)
204.	6	5	τ CC(45)

ʊ- stretching, ʊss-symmetric stretching, ʊass- asymmetric stretching, δ- in-plane bending, γ-out-of-plane bending, τ-twisting, σ-rocking, ρ-scissoring, ω-wagging.

Table S4: Definition of internal coordinates of NHDIPSA

No	Symbol	Type	Definitions
**Stretching**			
1	ν_1	N-H	N63-H64
2	ν_2	O-H (hydroxyl)	O38-H39
3-24	ν_3–ν_{24}	C-H (aromatic)	C7-H16, C8-H15, C9-H14, C10-H13, C11-H12, C18-H27, C19-H26, C20-H25, C21-H24, C22-H23, C30-H37, C31-H36, C32-H35, C33-H34, C41-H49, C42-H48, C44-H47, C45-H46, C54-H59, C55-H60, C57-H62, C58-H61
25 – 26	υ_{ass} - $\upsilon_{ass\ (25-26)}$	$C-H_3$ (asymmetric)	C67-H69, C67-H70
27	$\upsilon_{ss\ (27)}$	$C-H_3$ (symmetric)	C67-H68
28	ν_{28}	C=O(carbonyl)	C65-O66
29	ν_{29}	C-O(hydroxy)	C29-O38
30	ν_{30}	N=C(imine)	N4-C3
31-36	ν_{31}–ν_{36}	N-C	N4-C5, N2-C3, N2-C1, N2-C40, N63-C56, N63-C65
37-40	ν_{37}–ν_{40}	C-C (aromatic inter ring)	C3-C28, C1-C6, C5-C17, C65-C67
41	ν_{41}	C=C	C5-C1
42-47	ν_{42}–ν_{47}	C-C (ring1)	C6-C7, C7-C8, C8-C9, C9-C10, C10-C11, C11-C6
48-53	ν_{48}–ν_{53}	C-C (ring2)	C17-C18, C18-C19, C19-C20, C20-C21, C21-C22, C22-C17
54-59	ν_{54}–ν_{59}	C-C (ring3)	C28-C29, C29-C30, C30-C31, C31-C32, C32-C33, C33-C28
60-65	ν_{60}–ν_{65}	C-C (ring4)	C40-C41, C41-C42, C42-C43, C43-C44, C44-C45, C45-C40
66-71	ν_{66}–ν_{71}	C-C (ring5)	C53-C54, C54-C55, C55-C56, C56-C57, C57-C58, C58-C53
72-73	ν_{72}-ν_{73}	S-C	S50-C43, S50-C53
74-75	ν_{74ass}–ν_{75ss}	S=O(sulfonyl)	S50-O51, S50-O52
Bending			
76	δ_{76}	C-N-H (in-plane)	C56-N63-H64
77	ω_{77}	C-O-H (wagging/)	C29-O38-H39
78	δ_{78}	N-C-N(in plane bending)	N2-C3-N4

79-100	δ_{79}–δ_{100}	C-C-H (in-plane bending/aromatic CH)	C6-C11- H12, C9-C10-H13, C8-C9-H14, C7-C8-H15, C6-C7-H16, C17-C22-H23, C20- C21-H24, C19-20-H25, C18-C19-H26, C17-C18-H27, C28-C33-H34, C31-C3-H35, C30-C31-H36, C29-C30-H37, C40-C45-H46, C43-C44-H47, C41-C42-H48, C40-C41-H49, C53-C54-H59, C54-C55-H60, C57-C58-H61, C56-C57-H62
101-103	ρ_{101}–ρ_{103}	H-C-H (methyl-scissoring)	H68-C67-H69, H68-C67-H70, H69-C67-H70
104-109	δ_{104}–δ_{109}	C-N-C(ring/in-plane)	C1-N2-C3, C3-N4-C5, C56-N63-C65, C1-C5-N4, C28-C3-N2, C67-C65-N63
110	δ_{110}	C-O-C (in-plane)	C28- O38-C29
111	δ_{111}	O-S-O (scissoring)	O51-S50-O52
112-115	δ_{112}–δ_{115}	S-C-C/C-C-S	S50-C53-C54, S50-C43-C44, C59-C53-S50, C42-C43-S50
116-119	δ_{116}–δ_{119}	O-S-C(in-plane)	O51-S50-C53, O52-S50-C53, O51-S50-C43, O52-S50-C43
120-134	δ_{120}–δ_{134}	C-C-C (ring internal in-plane bends)	C6-C7-C8, C8-C9-C10, C10-C11-C6, C17-C18-C19, C19-C20-C21, C21-C22-C17, C28-C29-C30, C30-C31-C32, C32-C33-C28, C40-C41-C42, C42-C43-C44, C44-C45-C40, C53-C54-C55, C55-C56-C57, C57-C58-C53
135-140	δ_{135}–δ_{140}	C-C-C (linking / non-ring)	C1-C6-C7, C1-C6-C11, C5-C17-C18, C5-C17-C22, C1-C5-C17, C5-C1-C6
Out of plane bending			
141	γ_{141}	C-H	C56-C65-N63-H64
142-163	γ_{142}- γ_{163}	C-H(Aromatic)	C6-C8-C7-H16, C7-C9-C8-H15, C8-C10-C9-H14, C9-C11-C10-H13, C10-C6-C11-H12, C17-C19-C18-H27, C18-C20-C19-H26, C19-C21-C20-H25, C20-C22-C21-H24, C21-C17-C22-H23, C29-C31-C30-H37, C30-C32-C31-H36, C31-C33-C32-H35, C40-C42-C41-H49, C41-C43-C42-H48, C43-C45-C44-H47, C44-C40-C45-H46, C53-C55-C54-H59, C54-C56-C55-H60, C54-C57-C58-H61, C58-C56-C57-H62, H62-C57-C58-H61,
164	ω_{164}	SO_2 (Wagging)	C59-C53-S50-O52
165	γ_{165}	C=O(Carbonyl wag)	O66-C65-N63-C67
Torsion			

166-180	τ_{166}- τ_{180}	C-C-C-H (aromatic)	C6-C7-C8-C9, C8-C9-C10-C11, C10-C11-C6-C7, C17-C18-C19-C20, C19-C20-C21-C22, C21-C22-C17-C18, C28-C29-C30-C31, C30-C31-C32-C33, C32-C33-C28-C29, C40-C41-C42-C43, C42-C43-C44-C45, C44-C45-C40-C41, C53-C54-C55-C56, C55-C56-C57-C58, C57-C58-C53-C54
181	τ_{181}	Methyl (CH_3)	H68–C67–C65–O66
182-187	τ_{182}- τ_{187}	C-C-C-C (Inter-ring)	C1-C6-C7-C11, C5-C17-C18-C22, C1-C5-C17-C18, C5-C1-C6-C11, C3-C28-C29-C33, C6-C1-C5-C17
188	τ_{188}	C-N-C-N (conjugated N)	C1-N2-C3-N4.
189	τ_{189}	N-C-C -N	N2-C1-C5-N4.
190 - 192	τ_{190}– τ_{192}	N-C-C-H	N2-C40-C41-H49, N63-C56-C57-H62, N63-C65-C67-H69
193	τ_{193}	C-C-C-S	C41-C42-C43-S50
194-195	τ_{194}- τ_{195}	S-C-C-H	S50-C43-C44-H47, S50-C43-C42-H48
196-199	τ_{196}- τ_{199}	C-C-S-O	C42-C43-S50-O51, C44-C43-S50-O51, C54-C53-S50-O51, C58-C53-S50-O51
200-201	τ_{200}- τ_{201}	C-C-N-H (Amide)	C55-C56-N63-H64, C57-C56-N63-H64. 1
202	τ_{202}	C-N-C-O	C56-N63-C65-O66
203	τ_{203}	H-N-C-O	H64-N63-C65-O66
204	τ_{204}	C-C-C-S torsion	C42–C43–S50–O51

ν- stretching, ν_{ss} – symmetric stretching, ν_{ass}- asymmetric stretching, δ - In-plane bending, γ- Out-of-plane bending, τ- Torsion, ω- wagging, ρ-Scissoring
